# *Candida auris MNN1* contributes to human neutrophil evasion via mechanisms independent of β-glucan unmasking

**DOI:** 10.1128/mbio.01701-26

**Published:** 2026-09-24

**Authors:** Ashley M. Holt, Mark V. Horton, Chad J. Johnson, Robert Zarnowski, Por Choua Vang, Megan Dean, Arianna Kaas, Brody D. Andes, Ryley Jones, Douglas Lowman, Michael D. Kruppa, Zuchao Ma, David L. Williams, Jeniel E. Nett

**Affiliations:** 1 Department of Medicine, University of Wisconsin-Madison 5228, Madison, Wisconsin, USA; 2 Department of Medical Microbiology & Immunology, University of Wisconsin-Madison 5228, Madison, Wisconsin, USA; 3 Department of Surgery, Quillen College of Medicine, Center for Inflammation, Infectious Disease, and Immunity, East Tennessee State University 4154, Johnson City, Tennessee, USA; 4 Department of Biomedical Sciences, Quillen College of Medicine, Center for Inflammation, Infectious Disease, and Immunity, East Tennessee State University 4154, Johnson City, Tennessee, USA; The Lundquist Institute, Torrance, California, USA

**Keywords:** *Candida auris*, *Candidozyma auris*, mannosylation, mannan, cell wall, neutrophil, innate immunity, Dectin-2, Mincle, glycan recognition

## Abstract

Systemic *Candida* (*Candidozyma*) *auris* infections can have mortality rates up to 60%, and little is known about immune responses to this fungal pathogen. Neutrophils play a major role in immunity to many fungal pathogens, including *Candida* species. However, neutrophils internalize *C. auris* at a lower rate than *C. albicans*. We previously found that the *C. auris* cell wall mannan blocks access to β-glucan, an immunostimulatory motif. Here, we analyzed the immune-evasion properties of *C. auris* mannan separate from glucan masking. We generated eight mannosylation mutants and identified two with increased rates of phagocytosis by human neutrophils. One of these mutants, *mnn1*Δ, did not exhibit increased exposure of β-glucan. Nuclear magnetic resonance analysis of *mnn1*Δ mannan showed decreased α-1,3-mannose linkages and acid-labile side chains. These structural changes increased the affinity of human Dectin-2 and Mincle for mannans *in vitro*, and Fab blocking of Dectin-2 decreased human neutrophil phagocytosis of *mnn1*Δ. Overall, our work provides insight into human neutrophil recognition of *C. auris* mannans and further understanding of mechanisms of immune evasion for this fungal pathogen.

**IMPORTANCE:**

Cell surface glycan recognition plays an important role in the innate immune response to fungal pathogens. Here, we use *Candida auris* mannosylation mutants to identify *C. auris* genes that contribute to its neutrophil evasion properties. We identify a mannosylation gene, *MNN1*, that contributes to human and murine peripheral blood neutrophil evasion without significantly impacting beta-glucan masking. We attribute *MNN1* to the alteration of cell surface mannans that leads to decreased Dectin-2-binding affinity. While mannan has been associated with masking β-glucan in the *C. auris* cell wall, the impact of specific mannan structures on receptor binding presents an additional mechanism of neutrophil evasion for *C. auris*.

## INTRODUCTION

*Candida* (*Candidozyma*) *auris* is a recently emergent fungal pathogen that has caused outbreaks across the world. *C. auris* invasive infections are associated with mortality as high as 60%, and in some regional settings, *C. auris* bloodstream infections have become more common than *Candida albicans* bloodstream infections (1–6). In the United States, the number of *C. auris* clinical cases has increased approximately twofold annually in recent years (7, 8). *C. auris* isolates exhibit high rates of resistance to antifungals, with more than 90% of strains resistant to fluconazole and greater than 20% resistant to multiple drugs. In addition, pan-resistance to the three most common antifungal classes (azoles, echinocandins, and polyenes) has been reported (1, 9, 10). However, even with appropriate antifungal therapy, mortality remains high (1). Therefore, it is important to understand *C. auris* mechanisms of pathogenesis, including its interplay with host immunity.

Neutrophils are innate immune cells that play a critical role in protection against many fungal pathogens (11, 12). Compared to other *Candida* species, *C. auris* evades human neutrophil recognition and killing (13, 14). This *C. auris*-neutrophil interaction is mediated, in part, by the fungal cell wall structure. The outermost mannan layer functions to mask the inner immunostimulatory β-glucan (13–21). However, less is known about the direct interactions of the cell wall mannans with neutrophil receptors. Recent work has described distinct features of *C. auris* mannan when compared to *C. albicans*, including a lower molecular mass and unique structural moieties (15, 22, 23).

Here, we aimed to discern what mannan characteristics contribute to evasion of human neutrophil responses beyond β-glucan masking. We generated *C. auris* mutants with predicted disruption of N-linked mannan side chains and examined their impact on human neutrophil phagocytic responses. Of the mutants with increased human neutrophil phagocytosis, only one mutant, *mnn1*Δ, did not demonstrate increased cell surface β-glucan exposure. We show that *MNN1* contributes to neutrophil evasion through the alteration of cell wall mannans which in turn diminishes Dectin-2 binding and fungal recognition.

## RESULTS

### Disruption of *C. auris MNN1* increases susceptibility to human neutrophil phagocytosis and killing independent of glucan unmasking

**Fig 1:**
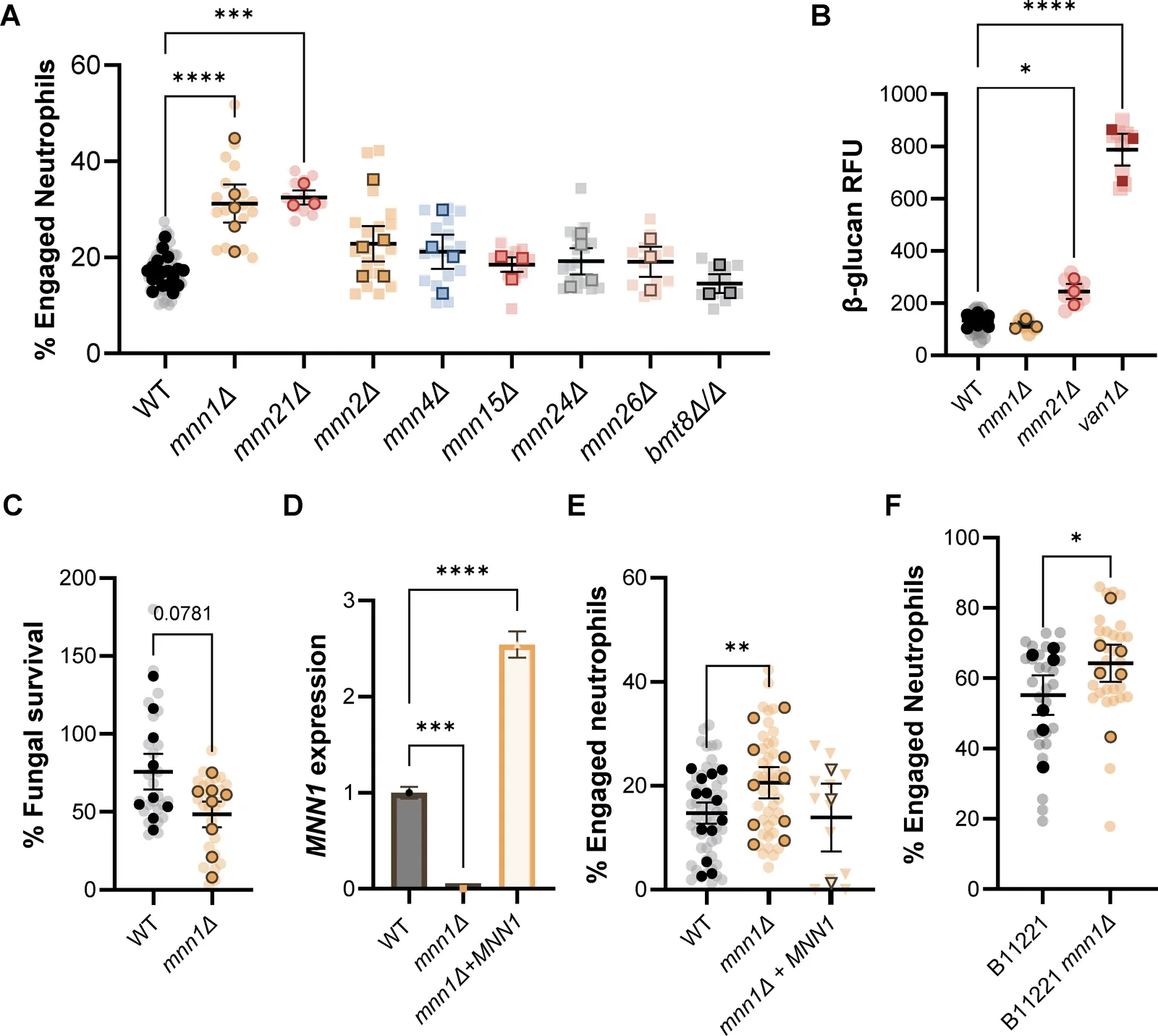
Primary human neutrophils are more likely to engage *C. auris mnn1*Δ compared with *C. auris* wild type. (**A**) *C. auris* mutants (with parent strain B11203) were stained with calcofluor white and incubated for 1 h with primary human neutrophils at a multiplicity of infection (MOI) of 4. Using fluorescence microscopy, the number of neutrophils engulfing yeast cells was enumerated, and the percentage of engaged neutrophils was calculated. (**B**) Cell surface β-glucan was labeled using rhFc:mDectin-1 protein conjugate with Alexa Fluor 488-conjugated anti-human IgG Fc antibody and quantified via plate reader measurements, with values adjusted to subtract the value of no primary controls. (**C**) *C. auris* strains were cultured with human neutrophils for 2 h, then neutrophils were lysed, and the inoculum was plated on yeast-peptone-dextrose (YPD) for viable burden calculation via colony-forming unit (CFU) enumeration. (**D**) RNA was isolated from each strain, and RT-PCR was performed. Expression of each gene is presented relative to that of *ACT1*. (**E**) Human neutrophils were labeled with calcein-AM and cultured for 1 h with individual *C. auris* strains labeled with calcofluor white. Fluorescent images were analyzed as in panel A. (**F**) Primary human neutrophils and fungal cells were stained and coincubated as in panel A. Human neutrophil phagocytosis of the strains was determined with epifluorescence microscopy as in panel A. For all experiments, *n* ≥ 3 replicates. Translucent points show all technical replicate values, and opaque/outlined points show biological replicate values. Lines indicate the mean of the biological replicates, with SEM shown as error bars. *P* value shown in panel **C** and *P < 0.05, **P < 0.01, ***P < 0.001, ****P < 0.0001 by (**A, B, and D**) ordinary one-way ANOVA of biological replicate values with Holm-Sidak multiple comparisons to *C. auris* wild type, (**C and F**) Wilcoxon test, or (**E**) mixed-effects analysis of biological replicate mean values with Holm-Sidak multiple comparisons to *C. auris* wild type.

To identify specific *C. auris* mannan residues important for neutrophil evasion, we constructed mutants with disruption of predicted mannosyltransferase genes based on data in the Candida Genome Database (http://www.candidagenome.org) (24). We selected genes with putative involvement in building *C. auris* mannan side chains (Table S1). We disrupted homologs in the *MNN1* family (*mnn1*Δ and *mnn15*Δ) with predicted α-1,3-mannosyltransferase activity (25–27), the *MNN2* family (*mnn2*Δ, *mnn21*Δ*, mnn24*Δ*,* and *mnn26*Δ) with predicted α-1,2-mannosyltransferase activity (28), the *BMT* family (*bmt8*Δ*/bmt1*Δ double knockout) with predicted β-1,2-mannosyltransferase activity (29, 30), and the *MNN4* family (*mnn4*Δ) with predicted phosphomannosylation activity (31). We utilized the parent strain B11203 (AR0389) from clade I, a major circulating clade (32). We found two mutants (*mnn1*Δ and *mnn21*Δ) to be significantly more susceptible to phagocytosis by human neutrophils compared to the parent strain (Fig. 1A).

To determine whether the increase in phagocytosis could be due to increased glucan exposure, we measured available cell surface β-glucan and included *van1*Δ as a positive control based on prior work showing disruption of this predicted α-1,6-mannosyltransferase leads to high β-glucan exposure (13). Similar to the wild-type strain, live *mnn1*Δ displayed minimal cell surface β-glucan exposure as measured by binding of soluble murine Dectin-1 (Fig. 1B) (33). This minimal binding did not significantly change when the binding assay was performed at 37°C in RPMI medium, the conditions used in phagocytosis assays (Fig. S1A). The *mnn1*Δ strain also exhibited minimal binding to recombinant human Dectin-1 receptor and did not display altered chitin exposure (Fig. S1B and S1C). Similarly, Fab blocking of human Dectin-1 did not significantly change phagocytosis rates of *mnn1*Δ (Fig. S1D). In contrast to *mnn1*Δ, *mnn21*Δ exhibited heightened β-glucan exposure (Fig. 1B). Because we could not exclude glucan exposure as a mechanism for the increase in phagocytosis of *mnn21*Δ, we elected to investigate *mnn1*Δ to parse out recognition of *C. auris* mannan structures independent of β-glucan recognition.

We questioned whether the increase in human neutrophil phagocytosis of *mnn1*Δ led to enhanced fungal killing. After co-culture with human neutrophils, fungal survival of *mnn1*Δ was decreased by approximately 20% compared with the wild-type strain (Fig. 1D; mean change in survival = −19.74% [95% CI: −39.26%, −0.2172%], *P* = 0.0781 by Wilcoxon test). The strains grew similarly in the absence of primary human neutrophils (Fig. S2). We additionally considered whether disruption of *MNN1* would influence other human neutrophil responses. Similar to the wild-type strain, *mnn1*Δ induced minimal reactive oxygen species and did not promote the formation of neutrophil extracellular traps (Fig. S3A and S3B). In addition, both strains exhibited comparable inhibition by hydrogen peroxide, consistent with similar susceptibilities to oxidative stress (Fig. S3C). Complementation of a wild-type allele of *MNN1* into *mnn1*Δ restored transcript levels and reversed its human neutrophil phagocytosis phenotype (Fig. 1D and E). To determine the generalizability of our results to another *C. auris* strain, we generated *mnn1*Δ in B11221 (AR0383, clade III) and similarly found increased phagocytosis compared to its parent strain (Fig. 1F). Taken together, these findings show *MNN1* contributes to evasion of neutrophil phagocytosis through mechanisms independent of β-glucan unmasking.

### Disruption of *MNN1* increases phagocytosis by murine peripheral blood neutrophils

**Fig 2:**
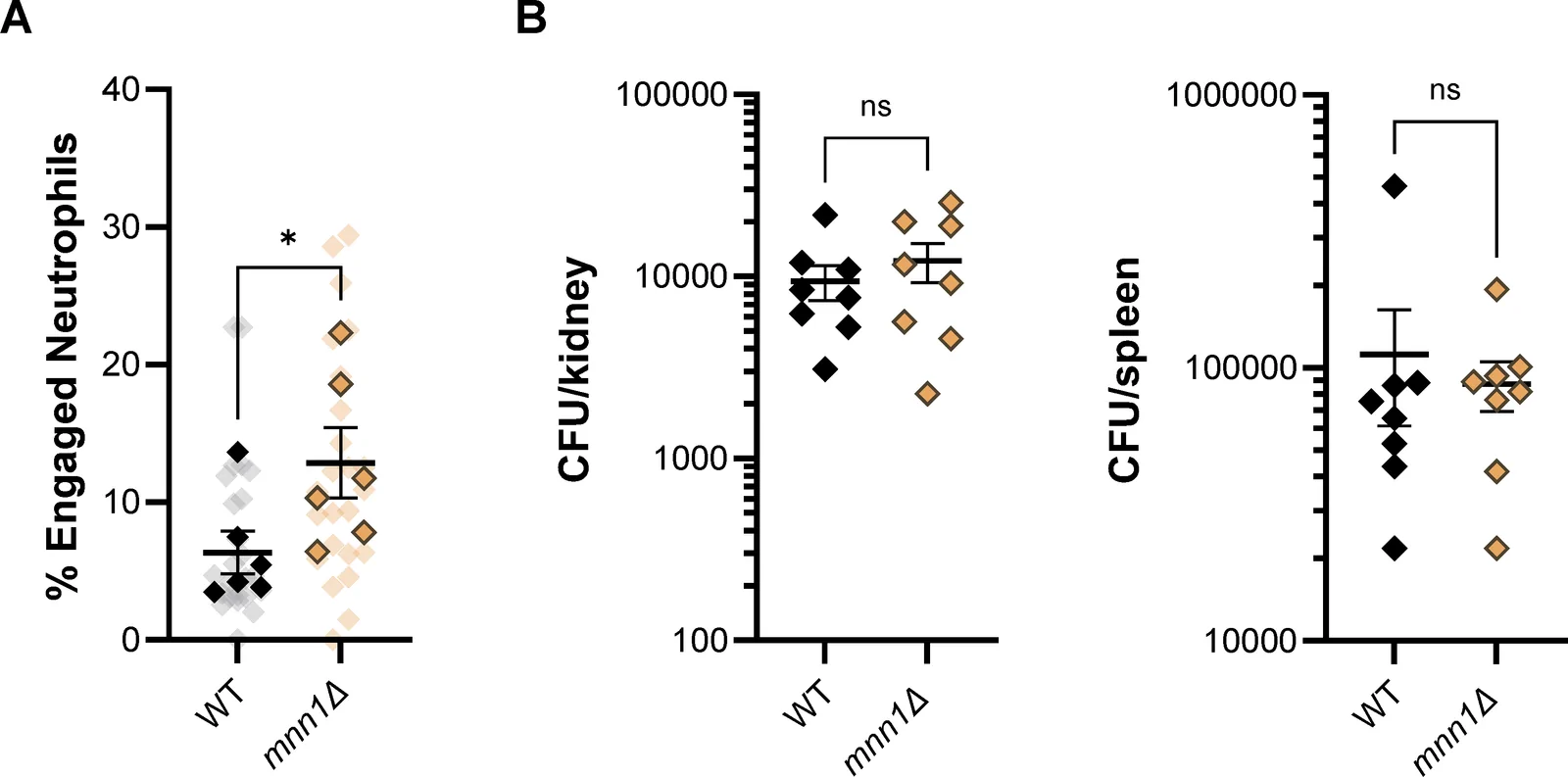
*C. auris mnn1*Δ shows increased murine peripheral blood neutrophil phagocytosis but no significant difference in a murine tail vein infection model. (**A**) Murine neutrophils were isolated from peripheral blood of female ICR mice. Neutrophils were incubated for 30 min with calcofluor white-stained fungal cells at an MOI of 4. Fluorescent images were analyzed for the number of neutrophils engaging yeast, and the percentage of engaged neutrophils was calculated. (**B**) *C. auris* strains (1 × 10^6^ cells) were injected into the tail veins of female ICR mice. Mice were sacrificed, and a kidney and spleen were collected from each animal at 48 h post-infection. The fungal CFU burden was quantified via plating on YPD. *n* ≥ 5. Translucent points show all technical replicate values. Mean of the biological replicates with SEM shown. *P < 0.05 by (**A**) Wilcoxon test or (**B**) Mann-Whitney test. ns = not significant.

Murine models are commonly used to assess *C. auris* infection and subsequent immune response *in vivo* (34, 35). To determine whether the observed *mnn1*Δ phenotype was relevant for mice, we isolated peripheral blood neutrophils from ICR mice and observed an approximately twofold increase in phagocytosis of *mnn1*Δ compared with wild type (Fig. 2A). Despite this difference in neutrophil response, we did not observe significant differences in spleen or kidney fungal burdens in a murine tail vein model of disseminated candidiasis without immune suppression (Fig. 2B). This finding may indicate that other cell types may compensate for the difference in neutrophil activities against wildtype and *mnn1*Δ in this model.

### Disruption of *MNN1* impacts mannan linkages without disrupting cell wall integrity

**Fig 3:**
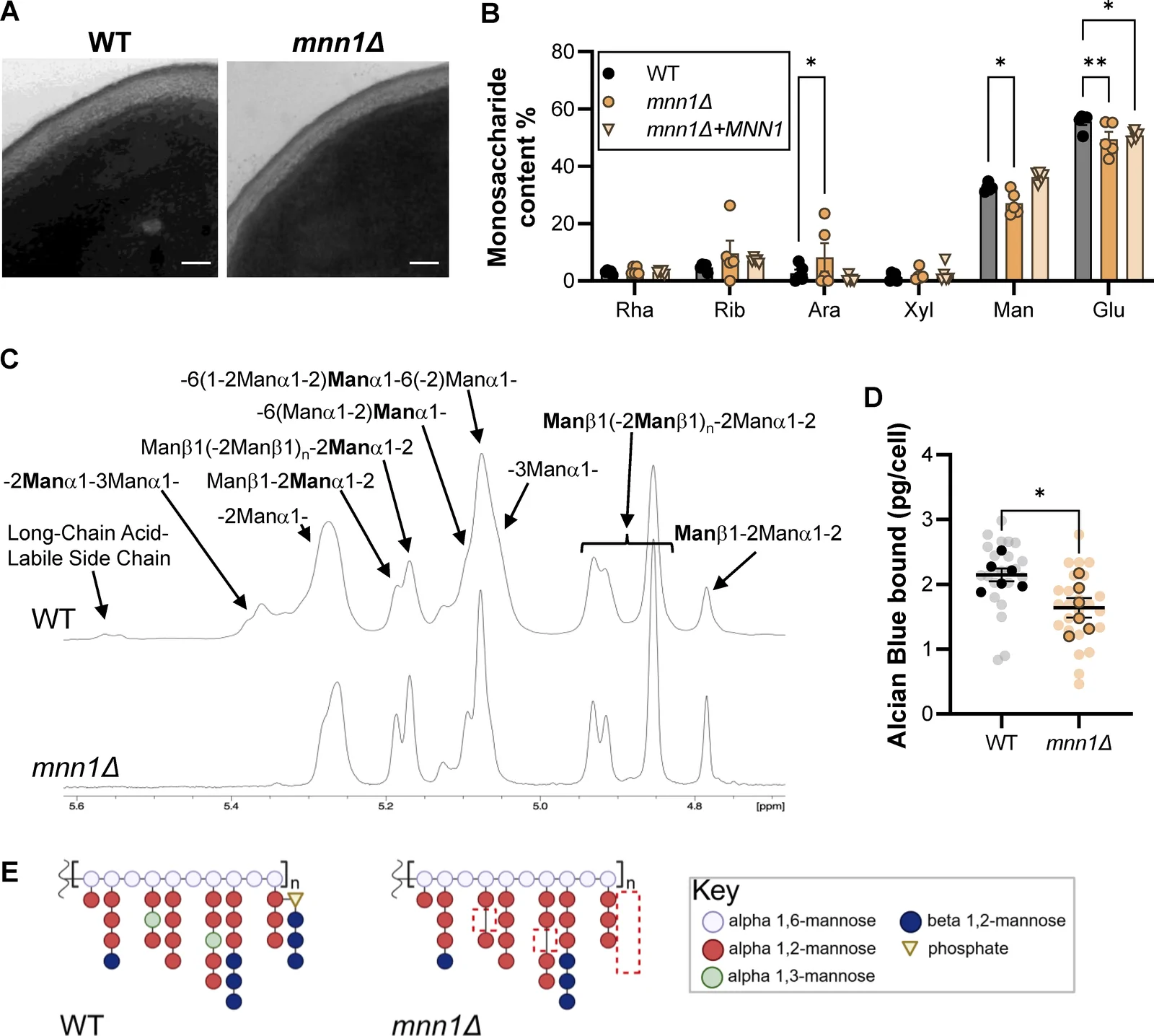
While preserving the overall cell wall architecture, *mnn1*Δ has modifications of the acid-labile side chains and α-1,3-mannose linkages in the cell wall mannans. (**A**) *C. auris* yeast were imaged via transmission electron microscopy at 213,000× magnification; scale bars represent 100 nm. (**B**) Monosaccharide composition of the cell walls was measured via gas chromatography. Rha, rhamnose; Rib, ribose; Ara, arabinose; Xyl, xylose; Man, mannose; Glu, glucose. (**C**) Proton nuclear magnetic resonance (^1^H NMR) spectra of mannans isolated from the cell walls of the strains were collected at 60°C, and the anomeric proton spectral region was plotted from 4.65 to 5.7 ppm. (**D**) Stationary-phase fungal cells were suspended in Alcian blue stain and incubated at room temperature (RT) for 10 min. Free Alcian blue stain was quantified via plate reader measurement of absorbance at 620 nm. Alcian blue bound to the cells was calculated in reference to a standard curve. (**E**) Predicted model of the changes to the *N*-linked mannan based on structural analysis. Created with BioRender. For all experiments, *n* ≥ 3 biological replicates. Translucent points show all technical replicate values. Lines indicate the mean of the biological replicates, with SEM shown as error bars. *P < 0.05, **P < 0.01 by (**B**) two-way ANOVA with Holm-Sidak multiple comparisons to *C. auris* wild type or (**D**) two-tailed unpaired *t*-test.

To gain an overall view of the *C. auris* cell wall architecture of *mnn1*Δ, we utilized transmission electron microscopy. We observed no visible changes to the general cell wall structure or the mannan fibril length for *mnn1*Δ compared to the parent strain (Fig. 3A and Fig. S4A). We then performed gas chromatography monosaccharide analysis and observed a small (5%) drop in the total mannose content for *mnn1*Δ when compared to the wild-type strain, signifying an overall minimal drop in total cell wall mannosylation (Fig. 3B). Consistent with intact cell wall integrity, the growth of *mnn1*Δ was similar to wild type in the presence of the cell wall stressors hygromycin, tunicamycin, or sodium dodecyl sulfate (Fig. S4B) (13).

We next isolated cell wall mannans and found mannans from wild type to have a molecular weight (by weight average;, Mw) of 502 kDa, whereas *mnn1*Δ mannans had a Mw of 495 kDa (Table S2). NMR (1D ^1^H NMR, Fig. 3C and Table S3) revealed changes in the *mnn1*Δ mannan spectrum related to the absence of α-1,3-linked mannose residues relative to mannan isolated from wild type. The spectrum for the *mnn1*Δ mannan also showed the absence of resonances at 5.555 ppm consistent with a lack of long-chain acid-labile side chains associated with phosphomannan. A decrease in phosphomannan content was also supported by the decrease in Alcian blue binding that we observed for *mnn1*Δ compared with wild type (Fig. 3D). We also assessed hydrophobicity using a microbial attachment to hydrocarbons assay and did not observe significant differences between the strains (Fig. S4C). Based on these data, we generated a model of the mannan changes associated with disruption of *MNN1* (Fig. 3E).

### Dectin-2 plays a role in phagocytosis of *mnn1*Δ

With the mannan structural differences highlighted by the NMR analysis, we sought to identify their influence on mannan recognition receptors and selected Mincle and Dectin-2 based on their expression in human neutrophils and roles in immune responses to other *Candida* species (36–43). We first assessed the binding of the recombinant receptors to live yeast with flow cytometry and found recombinant human and murine Dectin-2 to bind to both wild-type and *mnn1*Δ fungal cells without a significant difference between the strains (Fig. S5). In contrast, we did not observe binding of Mincle to live yeast (data not shown). To assess the binding affinity of these receptors to *C. auris* mannans, we used biolayer interferometry. Mannans isolated from *mnn1*Δ exhibited about twofold to threefold increased affinity for recombinant human receptors Mincle and Dectin-2 compared with wild-type mannan (Table 1).

**TABLE 1:** Recombinant human Dectin-2 and Mincle show increased affinity for isolated *mnn1*Δ mannans^*a*^

Strain	rhMincle		rhDectin-2	
	*K*_*D*_ (95% CI) (nM)	Affinity % increase	*K*_*D*_ (95% CI) (nM)	Affinity % increase
WT	2.48 (2.19–2.76)	—^*b*^	2.32 (1.75–2.88)	—
*mnn1*Δ	0.830 (0–1.76)	198%	0.566 (0.197–0.934)	309%

^*a*^ Recombinant receptor binding measured with biolayer interferometry.

^*b*^ "—” indicates not applicable.

**Fig 4:**
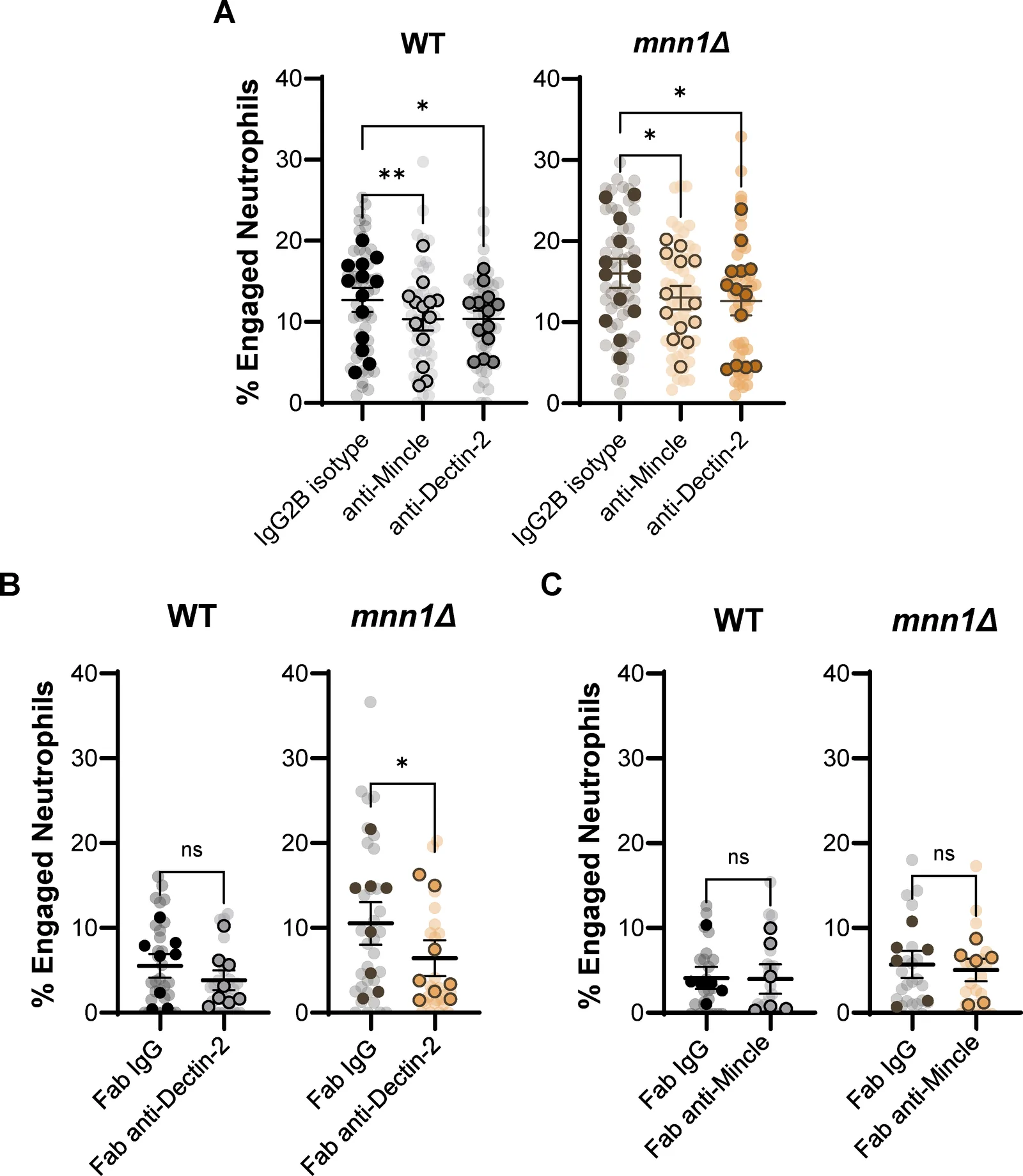
Receptor blockade (mAb and Fab) of human Dectin-2 decreases phagocytosis of *mnn1*Δ. (**A**) Prior to the phagocytosis assay, primary human neutrophils were treated with blocking antibodies against human Mincle or Dectin-2 or with IgG2B isotype antibody control (10 µg/mL for all antibody treatments) for 30 min on ice. After antibody treatment, neutrophils were co-incubated with calcofluor white-stained fungal cells for 1 h. Microscopy images were taken promptly after this incubation. Neutrophil engagement was quantified from microscopy images as described above. (**B and C**) Prior to the phagocytosis assay, primary human neutrophils were treated with Fabs generated from (**B**) mouse IgG2B or anti-human-Dectin-2 antibodies at approximately 5 µg/mL or (**C**) mouse IgG2B or anti-human-Mincle antibodies at approximately 1 µg/mL for 30 min at room temperature. After Fab treatment, neutrophils were co-incubated with calcofluor white-stained fungal cells for 1 h. Microscopy images were taken promptly after this incubation. Neutrophil engagement was quantified from microscopy images as described above. For the Fab experiment, ROUT outlier analysis with *Q* = 1% was performed on the engagement results from each image (technical replicate values), and outliers were removed before calculating each biological replicate mean. Data without outliers removed are shown in Fig. S6A and S6B. *n* ≥ 6. Points indicate the mean value of each biological replicate, with translucent points indicating all technical replicate values. *P < 0.05, **P < 0.01 by (**A**) repeated measures one-way ANOVA with Holm-Sidak multiple comparisons or (**B and C**) paired *t*-test to the isotype control. ns = not significant.

To study the functional impact of Dectin-2 and Mincle, we utilized blocking antibodies in *ex vivo* phagocytosis assays. Following treatment of primary human neutrophils with either anti-hMincle or anti-hDectin-2 monoclonal antibodies, we observed decreases in human neutrophil phagocytosis of both wild type and *mnn1*Δ (Fig. 4A). However, the rate of anti-Dectin-2-treated human neutrophil engagement of *mnn1*Δ (12.62 [95% CI: 8.712, 16.53]) was about the same as isotype-treated human neutrophil engagement of wild type (12.71 [95% CI: 9.476, 15.94]). To account for the potential contributions of Fc receptors when using full-length antibodies, we generated Fab fragments from the blocking antibodies. Following treatment with anti-hDectin-2 Fab, human neutrophil phagocytosis of *mnn1*Δ decreased, whereas phagocytosis of wild type did not significantly change (Fig. 4B, pre-outlier analysis data included in Fig. S6B). In contrast, anti-hMincle Fabs did not influence phagocytosis of either strain (Fig. 4C, pre-outlier analysis data included in Fig. S6C). The findings are consistent with the role of hDectin-2 in recognition and phagocytosis of *mnn1*Δ.

## DISCUSSION

Here, we demonstrate that small structural changes to the cell wall mannan layer of *C. auris* can alter human neutrophil recognition of this fungal pathogen. Previous reports have highlighted mannans as an important element in the interactions between *C. auris* and innate immune cells (13–15, 44). Currently, a well-defined role of *C. auris* mannan is the masking of inner immunostimulatory β-glucan. Using *C. auris* mutant strains with major disruptions in the cell wall mannan, our group and others have concluded that *C. auris* mannans function to mask β-glucans from recognition by innate immune cells, including human neutrophils and murine bone-marrow-derived macrophages (13, 14, 44). In contrast, the *mnn1*Δ mutant described here does not display major changes to cell wall architecture or composition and lacks glucan unmasking. The association of specific mannan moieties (phosphomannan side chains and α-1,3-mannose linkages) to neutrophil function is a newly elucidated mechanism for *C. auris* evasion of neutrophils and likely contributes to the resilience of *C. auris* in human disease.

Our studies focused on neutrophils as a subset of innate immune cells because of their clinical importance and frontline role in responses to candidiasis in patients (11, 12). We found mouse peripheral blood neutrophil responses to *mnn1*Δ to mirror human neutrophil responses. While there are distinct differences between human and murine neutrophils, we used peripheral blood neutrophils from human subjects and mice to best compare the function of terminally differentiated cells in the bloodstream, an important anatomical site for invasive candidiasis (45, 46). However, it is possible that murine neutrophils collected from the peritoneum or bone marrow may exhibit a different response to *mnn1*Δ due to a reliance on other receptors and/or a different activation status. Disruption of *MNN1* may also impact interaction with other cells, such as macrophages, perhaps through dectin-2 or mannose receptor (15, 23). Despite the observation of increased phagocytosis of *mnn1*Δ by both mouse and human neutrophils, we did not find that this correlated with a lower spleen or kidney fungal burden of *mnn1*Δ in a mouse model of intravenous candidiasis. In this context, macrophages or other cell types may contribute to recognition of *C. auris* independent of these mannan changes and compensate for the difference in neutrophil recognition of *mnn1*Δ and wild type. In contrast to the predominance of neutrophils in human blood, murine peripheral blood contains a lower number of neutrophils and exhibits a predominance of lymphocytes (45). Therefore, using murine models to understand the mechanisms for diseases in which neutrophils play a central role in human disease is not necessarily representative (45). Responses may also vary by mouse breed. We used ICR outbred mice to include some genetic diversity in an immunocompetent animal model. However, other studies have demonstrated differences in fungal virulence among inbred mouse strains, including work comparing *C. albicans* and *C. auris* (47–50).

Our studies of the *mnn1*Δ mannan structure in *C. auris* also highlight similarities and differences to the members of the *MNN1* mannosyltransferase family among *Candida* spp. *C. albicans* contains six genes in the *MNN1* gene family, predicted to be involved in α-1,3-mannosyltransferase activity in cell wall mannan (25–27). Different *Candida* species contain a varying number of *MNN1* family genes (25, 51), which may contribute to differences in mannosylation gene regulation and mannan structure across *Candida* species (22). When compared with *C. albicans* gene products via BLASTp analysis, *C. auris* Mnn1p has the closest homology to *C. albicans* Mnn14p (97% query cover, 37% identity) (52). Similar to *C. albicans mnn14*Δ, we noted a reduction in the α-1,3-mannose linkages in *C. auris mnn1*Δ. However, while *C. albicans mnn14*Δ has been shown to display increased phosphomannan side chains, we found that *C. auris mnn1*Δ has decreased phosphomannan side chains (25). We did not detect differences between *C. auris mnn1*Δ and wild type with conventional transmission electron microscopy. However, cryo-electron microscopy techniques have been demonstrated to be more sensitive for detection of differences (53). We did not find reports of electron imaging of *C. albicans mnn14*Δ using either technique or assessment of changes to cell surface glucan. Prior work has shown that disruption of *MNN14* in *C. albicans* does not influence cytokine production by human peripheral blood monocytes, but the effect of disruption on interactions with primary human neutrophils is unknown (25). Studies utilizing a BALB/c mouse model of disseminated infection found *C. albicans mnn14*Δ to have decreased virulence and tissue fungal burdens compared to wild type (25). This contrasts with our findings of *C. auris mnn1*Δ using an ICR mouse model of infection, where we did not observe significant differences in spleen and kidney fungal burdens between *C. auris mnn1*Δ and wild type. However, how *C. albicans MNN14* disruption impacts β-glucan masking and cell wall architecture is less clear, so it is difficult to parse out whether these changes could be influencing immune responses.

In this work, we examine the impact of *MNN1* disruption on binding of *C. auris* by known mannan pattern recognition receptors, Mincle and Dectin-2 (36–43). We did not detect binding of recombinant human Mincle to the surface of live fungal cells. This could be due to the need for receptor oligomerization to bind to cell surface structures, or Mincle may only bind glycolipid components located deeper in the cell wall that are made accessible following mannan isolation (54, 55). However, with monoclonal antibody blocking of hMincle and hDectin-2, we observed small decreases in primary human neutrophil phagocytosis of both *C. auris mnn1*Δ and wild type with a decrease from Dectin-2 blocking slightly larger for *mnn1*Δ than wild type. This corresponds with previous work, with murine bone-marrow-derived macrophages where the dynamics of *C. auris* phagocytosis were altered by knockouts of Mincle and Dectin-2 (56). However, with Fab blocking, we observed a decrease in human neutrophil phagocytosis of *mnn1*Δ with anti-hDectin-2 Fab but not with anti-hMincle Fab. Neither Fab treatment significantly impacted human neutrophil phagocytosis of *C. auris* wild type. These data show a greater role for hDectin-2 in human neutrophil recognition and phagocytosis of *mnn1*Δ than wild type.

Multiple human immune receptors are likely to contribute to mannan recognition of *C. auris* during an infection, but here, we identified one receptor, Dectin-2, with altered binding corresponding to the mannan structural differences with disruption of a single *C. auris* gene, *MNN1*. In our studies, the affinity of *in vitro* hDectin-2 binding to *mnn1*Δ mannan more closely mimics its observed affinity for the wild-type *C. albicans* mannan in that it is much higher than wild-type *C. auris* mannans (15). We also demonstrate binding of recombinant hDectin-2 to the live intact cell surface of *C. auris* wild type and *mnn1*Δ. Prior work characterized Dectin-2 to bind core N-linked mannans located deeper in the *C. albicans* cell wall (43, 57). This aligns with additional structural characterization that suggests Dectin-2 binds to α-1,2-linked mannose dimers in internal positions within mannans and other glycans (58). We propose that decreased α-1,3-linked mannose and phosphomannan residues in the cell wall of *mnn1*Δ compared with wild type allow for greater affinity binding of Dectin-2 to α-1,2-linked mannose residues deeper in the mannan structures. Overall, this work builds on our understanding of immune recognition of *C. auris* mannans and points to mannan linkages that help *C. auris* decrease recognition via a specific immune receptor, Dectin-2.

## MATERIALS AND METHODS

### Strains and inoculum

All strains utilized in this study are listed in Table S4. *C. auris* isolate B11203 was used as the reference strain for generation of mutants due to a low rate of primary human neutrophil phagocytosis (13). All strains are stored in 15% glycerol, frozen at −80°C, and maintained by streaking onto YPD agar plates. Overnight cultures were grown in YPD liquid medium in an orbital shaker at 180 rpm at 30°C. Following overnight growth, fresh YPD broth was used to dilute *C. auris* cultures 1:10 before an additional 2-h growth period at 180 rpm at 30°C, unless otherwise specified. *C. auris* cultures were washed twice in Dulbecco’s phosphate-buffered saline (DPBS) and enumerated via hemocytometer.

### Generating mutants in *C. auris*

Mutant strains were generated using homologous recombination as previously described (13, 44, 59, 60). Briefly, gene deletion and subsequent complementation were achieved with a nourseothricin and/or hygromycin resistance marker-based strategy. Gene replacement cassettes were generated by amplification of genomic DNA from *C. auris* and the nourseothricin resistance gene from the pNAT plasmid (or hygromycin from pYM70 for double mutants) (61, 62). Deletion cassettes were generated via PCR using primers (Table S5) and transformed into *C. auris* via electroporation using a CRISPR-Cas9 system (13, 60). Plasmids pTO135 and pTO136 were generously shared by Dr. Teresa O’Meara (60). Appropriate integration was confirmed by PCR analysis.

### Collection of human neutrophils

As previously described, neutrophils were isolated via the Miltenyi Biotec MACSxpress Whole Blood Neutrophil Isolation Kit (Catalog # 130-104-434) (13, 63). Further removal of erythrocytes was performed using the MACSxpress erythrocyte depletion kit (Catalog # 130-098-196, Miltenyi Biotec, Inc., Auburn, CA). Neutrophils were suspended in RPMI 1640 (without phenol red) supplemented with glutamine (0.3 mg/mL) and 2% heat-inactivated fetal bovine serum (denoted as RPMI + 2% FBS).

### Human and murine peripheral blood neutrophil phagocytosis

*Candida* strains were fluorescently labeled with calcofluor white (100 µg/mL) for 10 min in the dark at RT (13, 63). Yeast cells were washed three times in DPBS, then added to a tissue-culture-treated 8-well microslide (Catalog # 80826, ibidi) at 4 × 10^5^ cells/well. Neutrophils were stained using calcein acetoxymethyl (AM) (Catalog # C34852, Thermo Fisher Scientific, Waltham, MA) at 0.5 µg/mL for 10 min in the dark at RT. Neutrophils (1 × 10^5^) were added to each microslide well and incubated for 1 h at 37°C in 5% CO_2_. Images were acquired immediately with a 20× objective and 1.5× multiplier on a Nikon Eclipse Ti2 inverted microscope equipped with an ORCA-Flash 4.0 LT sCMOS camera, stage top TIZW series Neco incubation system (Tokai Hit), TI2-S-SS-E motorized stage, and NIS-Elements imaging software. DIC, fluorescein isothiocyanate (FITC) (EX 466/40: EM 525/50), and DAPI (49,6-diamidino-2-phenylindole) (Ex 378/52: Em 447/60 nm) channels were captured for each image. Neutrophils engaging *C. auris* were defined as neutrophils that co-localized with fungal cells by fluorescence on epifluorescent images, with neutrophil fluorescence surrounding the fungal cell (examples in Fig. S7), and reported as the percent engaged neutrophils (engaged neutrophils in frame/total neutrophils in frame) × 100 (13).

### Labeling cell wall polysaccharides via immunofluorescence

β-Glucan unmasking was quantified using methods previously described (13), based on methods from Nogueira et al. (64). *C. auris* yeast at 1 × 10^7^ cells/mL were resuspended in 1 mL of 4°C blocking solution (5% heat-inactivated fetal bovine serum, 0.5% bovine serum albumin [BSA], 5 mM EDTA, and 2 mM NaN_3_ in 1× DPBS) and incubated on a MACSmix tube rotator (Miltenyi Biotec, Inc., Auburn, CA) at 9 rpm for 30 min at RT. Cells were rinsed twice with cold washing solution (0.5% BSA, 5 mM EDTA, and 2 mM NaN_3_ in DPBS) before labeling with recombinant murine dectin-1 protein conjugated to human Fc (Catalog # AG-40B-0138-C050, Adipogen) at 1 mg/mL in blocking solution on ice for 1 h. Blocking solution-only samples served as controls. After washing three times with washing solution, cells were suspended in a 1:200 dilution of Alexa Fluor 488-conjugated anti-human IgG Fc antibody (Catalog # 410705, BioLegend) in blocking solution. After incubation in the dark for 45 min on ice, samples were rinsed three times with washing solution and then adjusted to 1 × 10^7^ cells/mL in washing solution. Cell inoculum (150 mL) was added to wells of a black 96-well microtiter plate (Catalog # 3916, Costar) in triplicate, and fluorescence intensity was quantified in a microplate reader at 488/519 nm.

### Killing assay

Quantification of fungal killing by human neutrophils was performed via plating and CFU quantification (63) with modifications. Briefly, *C. auris* yeast (2 × 10^5^) and neutrophils (1 × 10^5^) were added to wells of a clear 96-well flat-bottom microtiter plate (Catalog # 351172, Corning) in triplicate and incubated for 2 h. Next, 10 µL of DNase I (10 mg/mL) was added to each well and incubated for 20 min at 37°C at 5% CO_2_ for cleavage of extracellular DNA. Contents of each well were removed and placed into individual 1.5 mL Eppendorf tubes, which were then vortexed and centrifuged for 2 min at 2,000 × *g*. The supernatant was removed, and neutrophils were lysed by resuspending in 600 µL with DNase I solution (100 ng/mL in ddH_2_O) and incubating for 20 min at 37°C at 5% CO_2_ twice. Wells of the 96-well microtiter plate were scraped twice to collect remaining yeast, and serial dilutions were plated onto YPD to calculate yeast survival.

### RNA isolation and quantitative RT-PCR

The RNeasy Protect Mini Kit (Catalog #74104, Qiagen) was used to isolate RNA from planktonic yeast cells, which was then quantified using a NanoDrop spectrophotometer. The QuantiTect Probe RT-PCR kit (Catalog #204443, Qiagen) was used to perform the quantitative RT-PCR with a CFX96 real-time PCR detection system (Bio-Rad) using the following program: 50°C for 30 min, initial denaturation at 95°C for 15 min, and then 40 cycles of 94°C for 15 s, and 60°C for 1 min using primers listed in Table S6 (13, 65). Reactions were performed in triplicate, and the expression of genes was analyzed in relation to *ACT1* using the ΔΔ*C*_*T*_ method (where *C*_*T*_ is the threshold cycle [66]).

### Animal maintenance

Specific-pathogen-free, female ICR/Swiss mice were used for all studies (Harlan Sprague-Dawley, Indianapolis, IN, USA). The animals were maintained in accordance with the criteria of the Association for Assessment and Accreditation of Laboratory Animal Care.

### Isolation of murine peripheral blood neutrophils

Murine peripheral blood was collected via axillary cutdown of female ICR mice. Neutrophils were isolated from the peripheral blood using STEMCELL Technologies EasySep Mouse Neutrophil Enrichment Kit (Catalog # 19762) as per manufacturer’s instructions. Neutrophils were counted via hemocytometer and resuspended to the desired concentration in RPMI + 2% FBS.

### Murine intravenous infection model

Infection was done as described previously (67). Briefly, *C. auris* were resuspended to 1 × 10^7^ cells/mL in sterile 0.9% saline. Disseminated infection was achieved by injection of 0.1 mL of inoculum via the lateral tail vein at time zero. After 48 h, the animals were sacrificed by CO_2_ asphyxiation, and kidneys and spleens of each mouse were harvested, homogenized, and plated for serial dilution enumeration.

### Transmission electron microscopy

Cell walls of *C. auris* strains were imaged via transmission electron microscopy, as reported previously (13). Yeast cells were fixed in 2% glutaraldehyde and 4% formaldehyde, postfixed with 1% potassium ferricyanide and 1% osmium tetroxide, and stained with 1% uranyl acetate. Samples were dehydrated in a series of ethanol solutions and embedded in Spurr’s resin. Sections of 70 nm were cut out and placed on copper grids, poststained with 8% uranyl acetate in Reynolds lead citrate and 50% methanol, and analyzed with a transmission electron microscope (Philips CM120).

### Cell wall monosaccharide analysis via gas chromatography

*C. auris* (1 mL of 5 × 10^7^ cells/mL) was diluted into DPBS in 2 mL micro-tubes (Sarstedt) containing glass beads and disrupted using a bead-beater (Mini-Beadbeater, Biospec Products) at full speed for 1-min increments for a total of 5 min with 1-min incubation on ice between disruptions. The disrupted cells were transferred to fresh 1.5 mL Eppendorf tubes, pelleted at 1200 × *g*, and rinsed six times with ddH_2_O. Cell walls were lyophilized and then hydrolyzed in 2 N trifluoroacetic acid (Sigma-Aldrich) at 120°C for 90 min. Cell wall carbohydrates were analyzed in the same manner as described (13).

### NMR analysis

Mannan was isolated from the two *C. auris* strains, in duplicate, as previously described (13, 15). The mannans were lyophilized and stored at −20°C until assayed. Isolates were dissolved in water and passed through a C18 reverse-phase column to remove lipids and proteins that co-extracted with the mannan. This resulted in mannan that was ~90% pure. Fractions were collected, dried under streaming air, and resuspended in deuterium oxide (D_2_O) prior to NMR analysis. NMR data acquisition and analysis are based on methods described by Horton et al. (13). In summary, ^1^H NMR spectra for mannan were collected on a Bruker Avance III 400 NMR spectrometer operating at 333°K (60°C) in 5 mm NMR tubes. Mannan (~7 mg) was dissolved in ~600 µL D_2_O (Cambridge Isotope Laboratories, 99.81% deuterated). Chemical shift referencing was accomplished relative to trimethylsilylpropionate at 0.0 ppm. NMR spectra were collected using the following parameters: 300 scans, 65,536 data points, 20.69 ppm sweep width centered at 6.175 ppm, and 1-s pulse delay. Spectra were processed using exponential apodization with 0.3 Hz line broadening. Data processing was accomplished using TopSpin (version 4.0.9).

### Alcian blue binding assays

Alcian blue binding was carried out as described (68). Cells in stationary growth phase were collected, washed twice with DPBS, and adjusted to 1.4 × 10^7^ cells/mL. Cells were pelleted by centrifugation and resuspended in 30 μg/mL Alcian blue (in 0.02 M HCl) and incubated at RT for 15 min. Then, the cell suspension was centrifuged, and the supernatant was collected for quantification of Alcian blue by absorbance at 620 nm. The concentration of the free (non-bound) dye was then determined by comparison to a standard curve (18.75–300 μg/mL) as previously described (68).

### Recombinant receptor binding affinity

The binding of recombinant Dectin-2 and Mincle receptors (Catalog # 3114-DC-050 and 8995-CL-050, respectively, R&D Systems) to isolated mannan was assessed as previously described (15). Increasing concentrations of the respective ligands (3.125–400 μg/mL) were used to generate saturation curves. Binding affinities were calculated for mannans isolated from the *C. auris* strains as previously described (69). The Ni-NTA biosensor was equilibrated for 3 min before being exposed for 10 min to 0.1 μg/mL His-tagged rhDectin-2 or rhMincle receptor proteins and dissociated for 10 min in 10× kinetics buffer to measure the BLI signal, consistently 20 s after transfer. This was followed by a series of eight 5-min exposures to an increasing concentration (twofold) of carbohydrate. A parallel biosensor with the immobilized receptors was placed in the 10× kinetics buffer without respective carbohydrate exposure to control for receptor dissociation. Results are reported as the dissociation constant *K*_*D*_ mean value with the 95% confidence interval.

### Antibody blockade or Fab blockade of human neutrophil receptors prior to neutrophil phagocytosis

Monoclonal antibody blockade was performed based on methods from Bruno et al. (15), with slight modifications. Prior to antibody or Fab treatment, neutrophils were stained using calcein AM (Catalog # C34852, Thermo Fisher Scientific, Waltham, MA) at 0.5 µg/mL for 10 min in the dark at RT. Neutrophils were pretreated with 10 µg/mL anti-Mincle, anti-Dectin-2, or control IgG2B (Catalog # mabg-hmcl-2, Invivogen; MAB31141 and MAB004, R&D Systems) for 30 min on ice. Fabs were isolated from full-length antibodies (mouse anti-hDectin-1, anti-hDectin-2 and IgG2B isotype control, MAB1859, MAB31141, and MAB004, R&D Systems; mouse anti-hMincle, mabg-hmcl-2, Invivogen) with Pierce Fab Preparation Kit according to the manufacturer’s protocol (Catalog # 44985, Thermo Scientific). Fab concentrations were approximated based on the mass of the antibody input and the output volume from the kit (anti-Dectin-1 and anti-Dectin-2 ~200 µg/mL; IgG2B ~ 175 µg/mL; anti-Mincle ~100 µg/mL). Fabs were diluted to twice the working concentration (10 µg/mL for Dectin-1 and Dectin-2; 2 µg/mL for Mincle, 10 and 2 µg/mL for IgG control; working concentration based on Fab titration) in RPMI +2% FBS, and 100 µL was combined with 100 µL of primary human neutrophils at 2 × 10^6^ cells/mL in RPMI + 2% FBS at RT for 30 min in a 96-well plate (Catalog # 3370, Co-Star). After antibody or Fab treatment, neutrophils were then added to a tissue-treated microslide ibidi device (Catalog # 80826, ibidi) with calcofluor white-stained *C. auris* yeast prior to a 1-h incubation at 37°C in 5% CO_2_, and microscopy and quantification of phagocytosis were done as described above.

### Statistical analysis

Statistical analysis was performed in GraphPad Prism 10. Statistical tests are reported in figure legends. ROUT outlier analysis (with *Q* = 1%) was performed on Fab blocking data as described in the figure legend (Fig. 4B and C), and the data are shown without outlier analysis in Fig. S6A and S6B.

## SUPPLEMENTAL MATERIAL

Supplemental Material mbio.01701-26-s0001.pdf Supplemental methods, tables, and figures.

ASM does not own the copyrights to Supplemental Material that may be linked to, or accessed through, an article. The authors have granted ASM a non-exclusive, world-wide license to publish the Supplemental Material files. Please contact the corresponding author directly for reuse.
