# Supplementary material for: Candida auris MNN1 contributes to human neutrophil evasion via mechanisms independent of β-glucan unmasking
Source: mBio. 2026 Sep 24;17(10):e01701-26. doi: 10.1128/mbio.01701-26 (PMC13712894; doi:10.1128/mbio.01701-26)
Supplement: Supplementary file 1 [file mbio.01701-26-s0001.pdf]

Supplemental Data

Supplemental Methods:

Chitin exposure assay. Chitin exposure data was similarly measured through utilizing wheat germ agglutinin conjugated to fluorescein isothiocyanate (WGA-FITC) to for adhesion to exposed chitin (1, 2). Briefly, *C. auris* strains were adjusted to 1 × 10^7 cells/ml and resuspended into 1 ml of 4°C DPBS containing 2% BSA (wt/vol). After incubating 30 min at room temperature (RT) using the MACSmix tube rotator (Miltenyi Biotec, Inc., Auburn, CA) at 9 rpm, *C. auris* yeast were collected via centrifugation and washed twice with ice cold DPBS containing 0.05% Tween 20 (vol/vol). Cells were labeled using 0.1 mg/ml WGA-FITC in tubes containing DPBS with 1% BSA (wt/vol). Unstained controls were included for each strain. Cells were incubated at RT in the dark for 1 h. Cells were washed three times with ice cold DPBS 0.05% Tween 20 and resuspended to 1 × 10^7 cells/ml in DPBS 0.05% Tween 20. Cell suspension (150 ml) was added in triplicate to wells of a black 96-well microtiter plate (Costar) and fluorescence intensity was quantified in a microplate reader at 488/519 nm.

Growth Curves. Yeast growth curve assays were performed as previously described (1). Yeast overnight cultures were rinsed two times with DPBS before counting with a hemocytometer and using RPMI + 2% FBS to resuspend at a concentration of 2 × 10^6 cells/ml. Wells of a clear 96-well microtiter plate were filled with 100ul of yeast solution in triplicate, then incubated with shaking for 20 h in a Bio Tek Synergy H1 microplate reader at 30°C. OD_{600} measurements were acquired each hour.

Sytox Green assay. Measurement of extracellular DNA was performed via Sytox Green staining as previously described (3). *C. auris* yeast and neutrophils were mixed together in at 2 × 10^5 cells/well in a black 96-well microtiter plate (Corning) for 4 h. Sytox Green was added to

quantify extracellular DNA via fluorescence (500/528nm), and wells containing phorbol myristate acetate (PMA [100 nM]) served as a positive control for eliciting free DNA.

Reactive oxygen species generation assay. Generation of reactive oxygen species by neutrophils was measured as previously described (1, 3). Briefly, the fluorescent oxidative stress dye CM-H_2DCFDA (Catalog # C6827, Life Technologies, Inc., Eugene, OR) was added to neutrophils in DPBS and incubated for 10 min in the dark. Neutrophils were rinsed twice with DPBS then added to 1:1 with *C. auris* strains in a black 96-well microtiter plate in triplicate (both at 2×10^5 cells/well). Acquisition of fluorescence measurements (495/527nm) was performed every 30 min for 4 h, with 100nM PMA added to a subset of wells as a positive control for comparison. Background fluorescence was calculated and subtracted from corresponding total fluorescence values prior to analysis.

Microbial attachment to hydrocarbons assay. The method for the microbial attachment to hydrocarbons assay was adapted from (4) with modifications. Overnight cultures were harvested by centrifugation and washed three times in PBS. The cells were standardized to an absorbance at 600 nm (A_{600}) of 0.4 in DPBS using a BioTek Synergy H1 microplate reader. Some of the cell suspension was reserved for an initial A_{600} reading (A0) to be measured simultaneously on the plate reader with the other samples after preparation. 1200 µL of each cell suspension in DPBS was transferred into clean, unused borosilicate glass tubes. 800 µL n-hexadecane (Catalog # A10322.AE, Thermo Scientific) was gently overlaid atop the culture suspension in the test tube. The tubes were allowed to rest for 10 minutes at RT, then capped and vortexed at max speed for 1 min to mix the hydrocarbon and aqueous phases. The tubes were left to rest for 15 minutes at RT to allow for separation of the phases, after which an aliquot of the lower aqueous layer was taken for the final A_{600} reading (A1) on the microplate reader.

The proportion of sequestered cells were calculated by dividing the final A_{600} reading (A1) by the initial reading (A0) according to the formula: [1 – (A1/A0)] * 100%.

Fungal growth when exposed to stressors. *C. auris* WT and *mnn1Δ* were screened for sensitivity to cell wall stressors by the microdilution method as previously reported (5). Briefly, overnight cultures were concentrated by centrifugation and washed three times with DPBS. Yeast cells were resuspended in YPD medium to $1x10^6$ cells/mL. 100μL of this suspension was incubated with cell wall stressing agents, across a range of doubling dilutions, for 16 h at 30°C, and the absorbance at 600nm was measured on a BioTek Synergy H1 microplate reader. The agents tested (maximum concentrations in parentheses) included hydrogen peroxide (0.4375%), SDS (0.4%), hygromycin B (1 mg/ml), and tunicamycin (10 mg/ml).

Gel permeation chromatography. The molecular weight, polydispersity, polymer distribution and Mark-Houwink (α) values were derived from gel permeation chromatography (GPC) as previously described (6, 7). The values were measured on a Viscotek/Malvern GPC system with a GPCMax autoinjector fitted to a TDA 305 detector (Viscotek/Malvern, Houston, TX). Three Waters Ultrahydrogel columns, i.e, 1200, 500 and 120, were fitted in series (Waters Corp. Milford, MA). The columns and detectors were maintained at 40°C within the TDA 305. The system was calibrated using Malvern pullulan and dextran standards. The mobile phase was 50mM sodium nitrite. The mobile phase was filter sterilized (0.45 μm into a 5 L mobile phase reservoir). Mannan samples were dissolved (3 mg/ml) in mobile phase (50 mM sodium nitrite, pH 7.3). The samples were incubated for ~60 min at 60°C, followed by sterile filtration (0.2 μm) and injected into the GPC (200 μL). Sample recovery was routinely > 98%. The data were analyzed using Viscotek OmniSec software v. 4.7.0.406. dn/dc was calculated using the OmniSec software (v. 4.6.1.354). The data were analyzed using a single peak assignment in order to obtain an average Mw for the entire polymer distribution. Replicate analysis of

calibration standards indicated reproducibility of + or - 3%, which is well within the limits of the technique.

Murine and human soluble recombinant Dectin-2 binding to live yeast cells. Dectin-2-Fc binding protocol was adapted from mDectin-1-Fc β-glucan unmasking quantification previously described (1), and based on methods from Nogueira et al (8). In brief, *C. auris* yeast at 1×10^7 cells/ml were resuspended in 1 ml of 4°C blocking solution (5% heat inactivated-fetal bovine serum, and 0.5% bovine serum albumin [BSA] in 1× made with 10x DPBS solution with magnesium and calcium, Fisher #J67746). Cells were incubated in blocking solution at 4°C for 1 hour. Cells were rinsed twice with cold washing solution (0.5% BSA in DPBS made with 10x DPBS solution with magnesium and calcium, Fisher #J67746) before labeling with recombinant murine or human dectin-2 protein conjugated to human Fc (Catalog # AG-40B-0139-C050, Adipogen or Catalog # 565004) at 10ug/ml in blocking solution on ice for 1 h. Inclusion of blocking solution-only samples served as controls. After washing three times with washing solution, cells were suspended in a 1:100 dilution of Alexa Fluor 488-conjugated anti-human IgG Fc antibody (Catalog # 410705, Biolegend) using blocking solution. After incubation in the dark for 1 hour on ice, samples were rinsed three times with washing solution and fixed with 2% formaldehyde in DPBS (without calcium and magnesium). Cells were then washed three times with DPBS and resuspended to 6.25×10^6 cells/ml in FACS buffer (DPBS with 2% FBS). Cells were stored at 4°C until quantification with flow cytometry within the next 24 hours after finishing cell preparation. Cells were analyzed on a ThermoFisher Attune NxT V6 Flow Cytometer with the gating strategy shown in Figure S5. Alexa Flour 488 flourescence was detected with a 488 blue laser and 530/30 detector filter with a 503LP dichroic filter. Analysis was done with FlowJo v10 (prior to June 11, 2026) and v11 (after June 11, 2026).

Table S1: Genes of interest identified in the Candida Genome Database

**Gene**	***C. auris* B8441 Gene ID #**
MNN1	B9J08_001669
MNN15	B9J08_004490
MNN2	B9J08_001935
MNN21	B9J08_003092
MNN24	B9J08_003847
MNN26	B9J08_004650
MNN4	B9J08_002229
BMT8	B9J08_003016
BMT1	B9J08_003051
VAN1	B9J08_003572

Table S2. Mean values for mannans isolated from *C. auris* wildtype and *mnn1Δ* measured with multi-detector gel permeation chromatography (6, 7).

	**WT**	***mnn1Δ***
**Peak RV (mL)**	23.588	23.455
**Mw (kD)**	502	495
**Mw/Mn**	1.301	1.385
**Rh(w) (nm)**	5.910	5.996
**Mark-Houwink (α)**	0.422	0.408

Table S3. ^{1}H-NMR chemical shifts and structural motifs in *C. auris mnn1Δ,* based on comparison of these 1D spectra with previous results.

Chemical Shift (ppm)	Structural Motif [a]
**5.555**	Long-chain acid-labile side chain
**5.38**	-2α**Man**1-3αMan1-
**5.362**	-2α**Man**1-3αMan1-
**5.048**	-2αMan1-3α**Man**1-
**Between 5.25 and 5.3 ppm**	-2αMan1-2α**Man**1-

[a] Repeat unit whose anomeric proton resonates at the designated chemical shift is bolded.

Table S4: Strains used in this study

Organism	Gene	Strain	Genotype	Source
C. auris	Reference	B11203	Clinical isolate (India)	(9), CDC
C. auris	*mnn1Δ*	RZU029	*mnn1Δ::NAT1*	This study
C. auris	*mnn15Δ*	RZU052	*mnn15Δ::NAT1*	This study
C. auris	*mnn1Δ+MNN1*	RZU0Y	*mnn1Δ::NAT1::MNN1/HYG*	This study
C. auris	*mnn2Δ*	RZU132	*mnn2Δ::NAT1*	This study
C. auris	*mnn21Δ*	RZU034	*mnn21Δ::NAT1*	This study
C. auris	*mnn24Δ*	RZU036	*mnn24Δ::NAT1*	This study
C. auris	*mnn26Δ*	RZU037	*mnn26Δ::NAT1*	This study
C. auris	*mnn4Δ*	RZU040	*mnn4Δ::NAT1*	This study
C. auris	*bmt8Δ/bmt1Δ*	RZU0X	*bmt8Δ::NAT1::bmt1Δ::HYG*	This study
C. auris		B11221	Clinical isolate (South Africa)	(9), CDC
C. auris	*mnn1Δ* B11221	AMH028	*mnn1Δ::NAT1*	This study
C. auris	*van1Δ*	MVH2	*van1Δ::NAT1*	(1)

CDC: Centers for Disease Control and prevention

Table S5: Primers used to construct *C. auris* mutant

Primer Name	Gene	Sequence
CR_MNN2 F1	*C. auris MNN2*	CCCACCAGCTCACATATCT
CR_MNN2 R1	*C. auris MNN2*	cacggcgcgcctagcagcggTACTCCACGTTCTTGAGAGG
CR_MNN2 F3	*C. auris MNN2*	gtcagcggccgcatccctgcAGGGCCTTAGAGATCTTAAAGA
CR_MNN2 R3	*C. auris MNN2*	GGGGACGTAAACCTCTTCA
CR_MNN2 nF	*C. auris MNN2*	GGCAGAAAGGGTATCATTCC
CR_MNN2 nR	*C. auris MNN2*	CCTCTCTCTGATCAAGTGGA
CR_MNN2 intF	*C. auris MNN2*	CTCCAAAGTCAGACAAGTGG
CR_MNN2 intR	*C. auris MNN2*	ACATCCAATCTGTCCAATGC
CR_MNN2 Nou UF	*C. auris MNN2*	CCAGGCTATTGTGAGGATTG
CR_MNN2 Nou UR	*C. auris MNN2*	TGGGGATGTATGGGCTAAAT
CR_MNN2 Nou DF	*C. auris MNN2*	CCCAGATGCGAAGTTAAGTG
CR_MNN2 Nou DR	*C. auris MNN2*	AAGTACAAGTCTATTCGCGC
CR_MNN1 F1	*C. auris MNN1*	TGTGGTGGTTTTCAATCAGG
CR_MNN1 R1	*C. auris MNN1*	cacggcgcgcctagcagcggCCAAGTAACGAAGCACCTTT
CR_MNN1 F3	*C. auris MNN1*	gtcagcggccgcatccctgcATCAAGTTCGAGAGGTACCC
CR_MNN1 R3	*C. auris MNN1*	TAGTTCATCATGTGCCAAGC
CR_MNN1 nF	*C. auris MNN1*	AAGGAATCTCTCACCCACAA
CR_MNN1 nR	*C. auris MNN1*	TACGGTTTCTATCGAACGGA
CR_MNN1 intF	*C. auris MNN1*	GTGCTACATTCAGTCACAGG
CR_MNN1 intR	*C. auris MNN1*	ACTGACATCATAAACGGCTG
CR_MNN1 Nou UF	*C. auris MNN1*	GTACGCCAGGTCCTATTACT
CR_MNN1 Nou UR	*C. auris MNN1*	GTATGGGCTAAATGTACGGG
CR_MNN1 Nou DF	*C. auris MNN1*	AAGTTAAGTGCGCAGAAAGT
CR_MNN1 Nou DR	*C. auris MNN1*	TTCCTTATAGTAGCCGCCAA
CR_MNN1 compl F1/ORF	*C. auris MNN1*	TCGTAAATTTTATCTGAATTGG
CR_MNN1 compl R1/ORF	*C. auris MNN1*	cacggcgcgcctagcagcggAATACAAAAAGAGGAAAATGCT
CR_MNN1 compl F3	*C. auris MNN1*	gtcagcggccgcatccctgcCCGGTAATAATTTTGTAATTTCT
CR_MNN1 compl R3	*C. auris MNN1*	TATATCCAAGATCTCGACTCAA
CR_MNN1 compl nF	*C. auris MNN1*	CAATGCCTCTTCCTACACTC
CR_MNN1 compl nR	*C. auris MNN1*	AAACGTATGAGTATTGGCTTCT
CR_MNN1 compl UF	*C. auris MNN1*	GTCCACTGCCCATTTGAT
CR_MNN1 compl UR	*C. auris MNN1*	CAAGGTATTTCCAGTAATAGGAC
CR_MNN1 compl DF	*C. auris MNN1*	TATTATTTTCGTCACGGGTAT
CR_MNN1 compl DR	*C. auris MNN1*	TATCCAAGATCTCGACTCAAA
CR_MNN15 F1	*C. auris MNN15*	AGATTTGCACTACTCCTCGT

CR_MNN15 R1	*C. auris MNN15*	cacggcgcgcctagcagcggACTGAAGAACTGTTTGCCAA
CR_MNN15 F3	*C. auris MNN15*	gtcagcggccgcatccctgcGTATCAAACCTGTCACGGAG
CR_MNN15 R3	*C. auris MNN15*	CATAGGATTGATAGGCGTCC
CR_MNN15 nF	*C. auris MNN15*	CAGAGAAGTTCGAGAAGACG
CR_MNN15 nR	*C. auris MNN15*	GCTCTGTCTTCTTCGTTGAA
CR_MNN15 intF	*C. auris MNN15*	TTTTGTCGAGGACCTGTTTC
CR_MNN15 intR	*C. auris MNN15*	GACATTGCCAACCAGTACAT
CR_MNN15 Nou UF	*C. auris MNN15*	TTGCTTGACGACTACCATTG
CR_MNN15 Nou UR	*C. auris MNN15*	CAAGACTGTCAAGGAGGGTA
CR_MNN15 Nou DF	*C. auris MNN15*	AAGTTAAGTGCGCAGAAAGT
CR_MNN15 Nou DR	*C. auris MNN15*	TGAACATGCCATCATCACTA
CR_MNN21 F1	*C. auris MNN21*	CTTTTGTCTCGCTGTCCTG
CR_MNN21 R1	*C. auris MNN21*	cacggcgcgcctagcagcggTTGAAAGCAAGATCGCAAG
CR_MNN21 F3	*C. auris MNN21*	gtcagcggccgcatccctgcAGACCATTTATTCAAGCACGA
CR_MNN21 R3	*C. auris MNN21*	ACTCTGTTGTCTTGAGAAATTCT
CR_MNN21 nF	*C. auris MNN21*	GATCATCACTGTGAAAACGC
CR_MNN21 nR	*C. auris MNN21*	TCGACGTTAAGATTGAGGTC
CR_MNN21 intF	*C. auris MNN21*	TCGATGACCTAAAAGCCTCT
CR_MNN21 intR	*C. auris MNN21*	CTGGATCGAATTGACCCATC
CR_MNN21 Nou UF	*C. auris MNN21*	TCTTTCTCAAGTCGACCGTA
CR_MNN21 Nou UR	*C. auris MNN21*	CAAGACTGTCAAGGAGGGTA
CR_MNN21 Nou DF	*C. auris MNN21*	AAGTTAAGTGCGCAGAAAGT
CR_MNN21 Nou DR	*C. auris MNN21*	TGCTCCATGAAAGTTACGAA
CR_MNN24 F1	*C. auris MNN24*	CCGGCGAAGTGGACTAGA
CR_MNN24 R1	*C. auris MNN24*	cacggcgcgcctagcagcggACTATACCATCTCCCTCCATG

CR_MNN24 F3	*C. auris MNN24*	gtcagcggccgcatccctgcCACTCTTGGTCTCTTCGTTT
CR_MNN24 R3	*C. auris MNN24*	GCAGAACCAAGTCTCAACAT
CR_MNN24 nF	*C. auris MNN24*	ACACTTCCCTCGTTTCCAAA
CR_MNN24 nR	*C. auris MNN24*	ACCATGAGTCTCCTTCTTCA
CR_MNN24 intF	*C. auris MNN24*	AGTACAAGAATGACAACGGC
CR_MNN24 intR	*C. auris MNN24*	CCTGACTCAAGAGGGGATAG
CR_MNN24 Nou UF	*C. auris MNN24*	ATAGCTCGCGTTGAAGAAAA
CR_MNN24 Nou UR	*C. auris MNN24*	TGGGGATGTATGGGCTAAAT
CR_MNN24 Nou DF	*C. auris MNN24*	AAGTTAAGTGCGCAGAAAGT
CR_MNN24 Nou DR	*C. auris MNN24*	GTTGTCACAAACTTTGCTCC
CR_MNN26 F1	*C. auris MNN26*	TTCTCTTGAACATCGCCATC
CR_MNN26 R1	*C. auris MNN26*	cacggcgcgcctagcagcggTTCAAGGGTAGAGCACAACT
CR_MNN26 F3	*C. auris MNN26*	gtcagcggccgcatccctgcACTTGCCTCTCAGTAATCCA
CR_MNN26 R3	*C. auris MNN26*	CACACTTTAGTCTGGTCTCG
CR_MNN26 nF	*C. auris MNN26*	GACTCCAACGTCTATACCAAC
CR_MNN26 nR	*C. auris MNN26*	AGCCCATCGAGATATCAAGT
CR_MNN26 intF	*C. auris MNN26*	TTTGACCAAAGAAGAGCTCG
CR_MNN26 intR	*C. auris MNN26*	CAATGGTTGTCTCCTGGAAG
CR_MNN26 Nou UF	*C. auris MNN26*	TCGATGCTGTTAGGAAGACT
CR_MNN26 Nou UR	*C. auris MNN26*	TGGGGATGTATGGGCTAAAT
CR_MNN26 Nou DF	*C. auris MNN26*	AAGTTAAGTGCGCAGAAAGT
CR_MNN26 Nou DR	*C. auris MNN26*	CATGAGCTTTAGGATCTTTCGA
CR_MNN4 F1	*C. auris MNN4*	CCTGATACAACCAGCCAATT
CR_MNN4 R1	*C. auris MNN4*	cacggcgcgcctagcagcggTTGACTTCGACTTTGCAACT
CR_MNN4 F3	*C. auris MNN4*	gtcagcggccgcatccctgcTAAGCAAGGTATCAGGACGT
CR_MNN4 R3	*C. auris MNN4*	GCTAAGAATGGCTCGAACTT

CR_MNN4 nF	*C. auris MNN4*	AACACTGACCAAGACTCACT
CR_MNN4 nR	*C. auris MNN4*	GGGCGAAAACTCCTTAGC
CR_MNN4 intF	*C. auris MNN4*	TTACTCACGATGATGGCAAC
CR_MNN4 intR	*C. auris MNN4*	GTCAATGAATCTGGCATCGA
CR_MNN4 Nou UF	*C. auris MNN4*	CATGTTCATGGGCATCAAAC
CR_MNN4 Nou UR	*C. auris MNN4*	CAAGACTGTCAAGGAGGGTA
CR_MNN4 Nou DF	*C. auris MNN4*	AAGTTAAGTGCGCAGAAAGT
CR_MNN4 Nou DR	*C. auris MNN4*	CATGAAGCCCAATGTATCGT
CR_BMT8 F1	*C. auris BMT8*	AGGCGCAGATACTAATACCA
CR_BMT8 R1	*C. auris BMT8*	cacggcgcgcctagcagcgg AATTTCTGGTGGTGCAAGTT
CR_BMT8 F3	*C. auris BMT8*	gtcagcggccgcatccctgc CAACGATGCAAGTTCTCAGA
CR_BMT8 R3	*C. auris BMT8*	CCACCAAGAAGATAGTGTCC
CR_BMT8 nF	*C. auris BMT8*	TACTACCACTTCGAGCATGT
CR_BMT8 nR	*C. auris BMT8*	ATCCTTTTCGATTGCAAGCT
CR_BMT8 intF	*C. auris BMT8*	GTATTTCGATGCACAGCTTC
CR_BMT8 intR	*C. auris BMT8*	AAGCGAAACAAATGACGAGA
CR_BMT8 Nou UF	*C. auris BMT8*	TTTCCCAATTGCAATGCCTA
CR_BMT8 Nou UR	*C. auris BMT8*	TGGGGATGTATGGGCTAAAT
CR_BMT8 Nou DF	*C. auris BMT8*	AAGTTAAGTGCGCAGAAAGT
CR_BMT8 Nou DR	*C. auris BMT8*	CCTGGCCCGAGTAATAGTAT
CR_BMT1 F1	*C. auris BMT1*	acttttacaattggcgggtt
CR_BMT1 R1	*C. auris BMT1*	cacggcgcgcctagcagcggATAGGAATGAAAACGCTGGC
CR_BMT1 F3	*C. auris BMT1*	gtcagcggccgcatccctgcTAAGTGAGGTTGCTAGGGAG
CR_BMT1 R3	*C. auris BMT1*	gtgtcttcgtgtctatgctc
CR_BMT1 nF	*C. auris BMT1*	cccgataagctcattgaagg
CR_BMT1 nR	*C. auris BMT1*	cagctctgattgtccgaatt
CR_BMT1 intF	*C. auris BMT1*	ccaggcaatttcactcacat
CR_BMT1 intR	*C. auris BMT1*	gggtctcaactttgtttcca
CR_BMT1 HygB UF	*C. auris BMT1*	ggctttcattttgttgcagt
CR_BMT1 HygB UR	*C. auris BMT1*	cggcacaattgaatagggaa
CR_BMT1 HygB DF	*C. auris BMT1*	tcgtcacgggtattttctct
CR_BMT1 HygB DR	*C. auris BMT1*	gagcctatgaaactacgctg
LF1_MNN1_CR_AMH	*C. auris* B11221 *MNN1*	TATCGCAACCATGGACCACT
LR1_MNN1_CR_AMH	*C. auris* B11221 *MNN1*	cacggcgcgcctagcagcggTGTATGGGCAATGGGGACTT
RF1_MNN1_CR_AMH	*C. auris* B11221 *MNN1*	gtcagcggccgcatccctgcAGGGTTGATGGTAGCTGCAT
RR1_MNN1_CR_AMH	*C. auris* B11221 *MNN1*	TGAACGCGTTAGATGCCTTG
NF1_MNN1_CR_AMH	*C. auris* B11221 *MNN1*	GCATCTTTAACTTTGAACAGGCC

NR1_MNN1_CR_AMH	*C. auris* B11221 *MNN1*	TTGCCCCAAGATGAAGTGCT
Seq_F_MNN1_CR_AMH	*C. auris* B11221 *MNN1*	CCTTGGCCCCTGTATCTGAA
Seq_R_MNN1_CR_AMH	*C. auris* B11221 *MNN1*	GCGTTGCCAAAACTTCGATG
sgRNA1_F_MNN1_CR_AMH	*C. auris* B11221 *MNN1*	gcagatctcctacgacaagaGTTTTAGAGCTAGAAATAGCAAG
sgRNA1_R_MNN1_CR	*C. auris* B11221 *MNN1*	tcttgtcgtaggagatctgcCTGGACGAGTCCGGATTC
TO19_F	sgRNA	GTAAAACGACGGCCAG
TO18_R	sgRNA	CAGGAAACAGCTATGAC
oTO142 Cau_sgRNA_rev	sgRNA	CTGGATGGCGGCGTTAG
oTO141 Cau_sgRNA_fwd	sgRNA	CGAGATAGATCGAAATACGC
Cas9_F	Cas9	ctcactgctcttccctccaa
Cas9_R	Cas9	tccgtcccaaaaccttctca

Table S6: Primers utilized for quantitative PCR

Gene	Primers	Probe
MNN1	Forward: 5’ – TGACTTGTCCAGACAGCATATT – 3’<br>Reverse: 5’ – CTTGCATCCACGAACCAAAC – 3’	5’ - /56-FAM/AGTTCGAGAGGTACCCAACACAGGA/36-TAMSp/ -3’
MNN11	Forward: 5’ – CAAGTGGGTTCGTCTCTACTG – 3’<br>Reverse: 5’ – GAGGTCGACATTCTGGTTCAT -3’	5’ - /56-FAM/TGGTACTTCGATCAGCACGGCTTT/36-TAMSp/ -3’
ACT1	Forward: 5’ – CCAGGGTATCATGGTTGGTATG – 3’<br>Reverse: 5’ – CCGTGCTCAATTGGGTATCT – 3’	5’ - /56-FAM/ACCGACATACGAATCCTTCTGGCC/36-TAMSp/ -3’

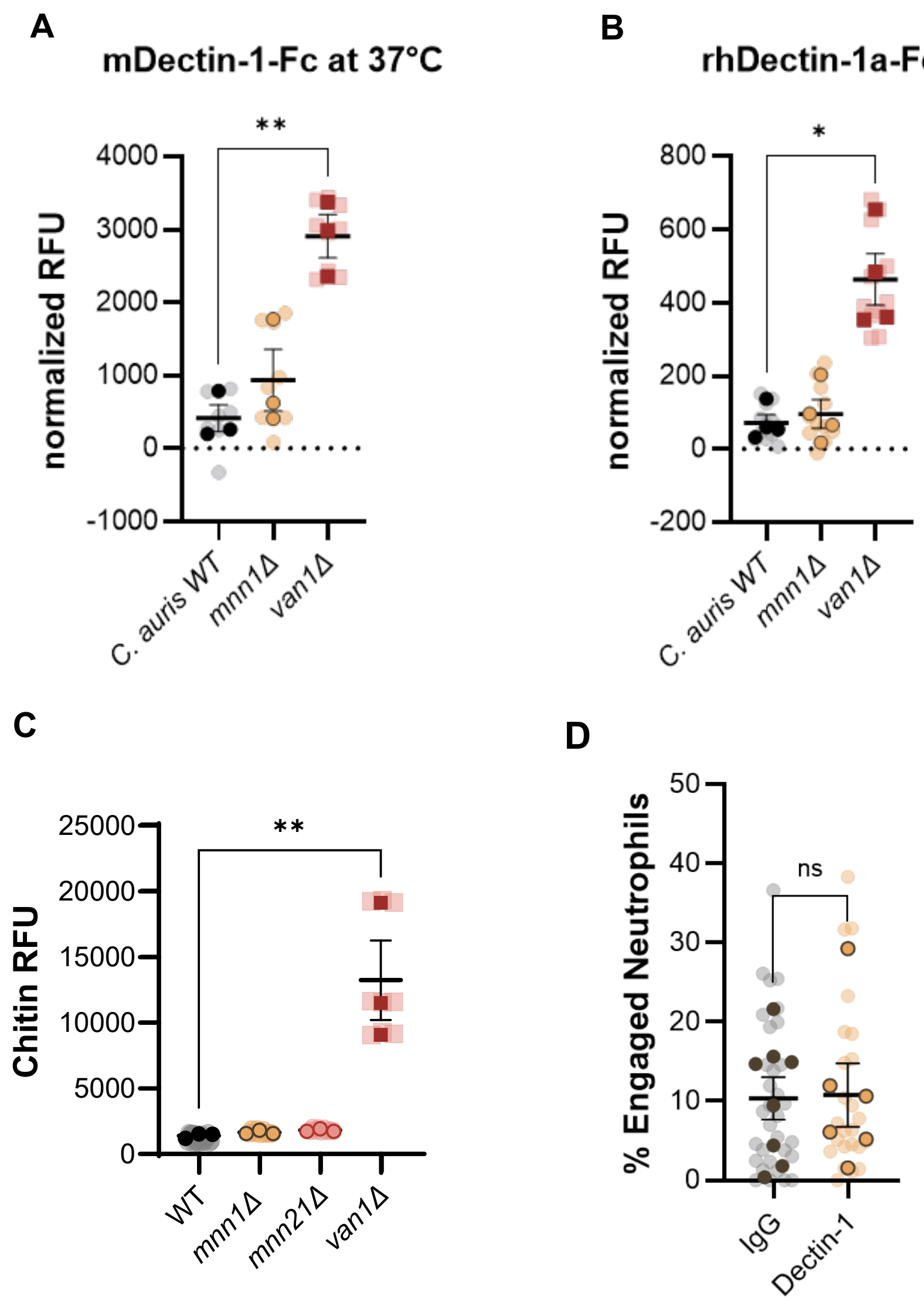

Figure S1. *C. auris mnn1Δ* does not exhibit significantly increased β-glucan exposure in conditions similar to the neutrophil engagement assay, and these mutants do not show increased binding of rhDectin-1 in comparison with wildtype. (**A**) Cell surface β-glucan was labeled at 37°C in RPMI+2%FBS using rhFc:mDectin-1 conjugate protein with Alexa Fluor 488-conjugated anti-human IgG Fc antibody and quantified via plate reader measurements with values adjusted to subtract the value of no primary controls. (**B**) Cell surface rhDectin-1a binding was assessed using rhDectin-1a-Fc conjugate protein with Alexa Fluor 488-conjugated anti-human IgG Fc antibody and quantified via plate reader measurements with values adjusted to subtract the value of no primary controls. (**C**) Cells were incubated with wheat germ agglutinin conjugated to fluorescein isothiocyanate (WGA-FITC), and binding was quantified via plate reader measurements with values adjusted to subtract the value of no primary controls. (**D**) Prior to the phagocytosis assay, primary human neutrophils were treated with Fabs generated from mouse IgG2B control or anti-human-Dectin-1 antibodies at approximately 5 µg/mL for 30 minutes at room temperature. After antibody treatment, neutrophils were co-incubated with calcofluor white-stained *mnn1Δ* cells for 1h. Microscopy images were taken promptly after this incubation. Neutrophil engagement was quantified from microscopy images as described above. For all experiments n≥3 biological replicates. Translucent points show all technical replicate values. Lines indicate mean of the biological replicates with SEM shown as error bars. *p<0.05, **p<0.01 by (A,B,C) ANOVA with Holm-Sidak multiple comparisons to *C. auris* wildtype or (D) paired t-test to the isotype control. ROUT outlier analysis (Q=1%) run on technical replicate values for data in (D), but no outliers identified. ns = not significant.

A

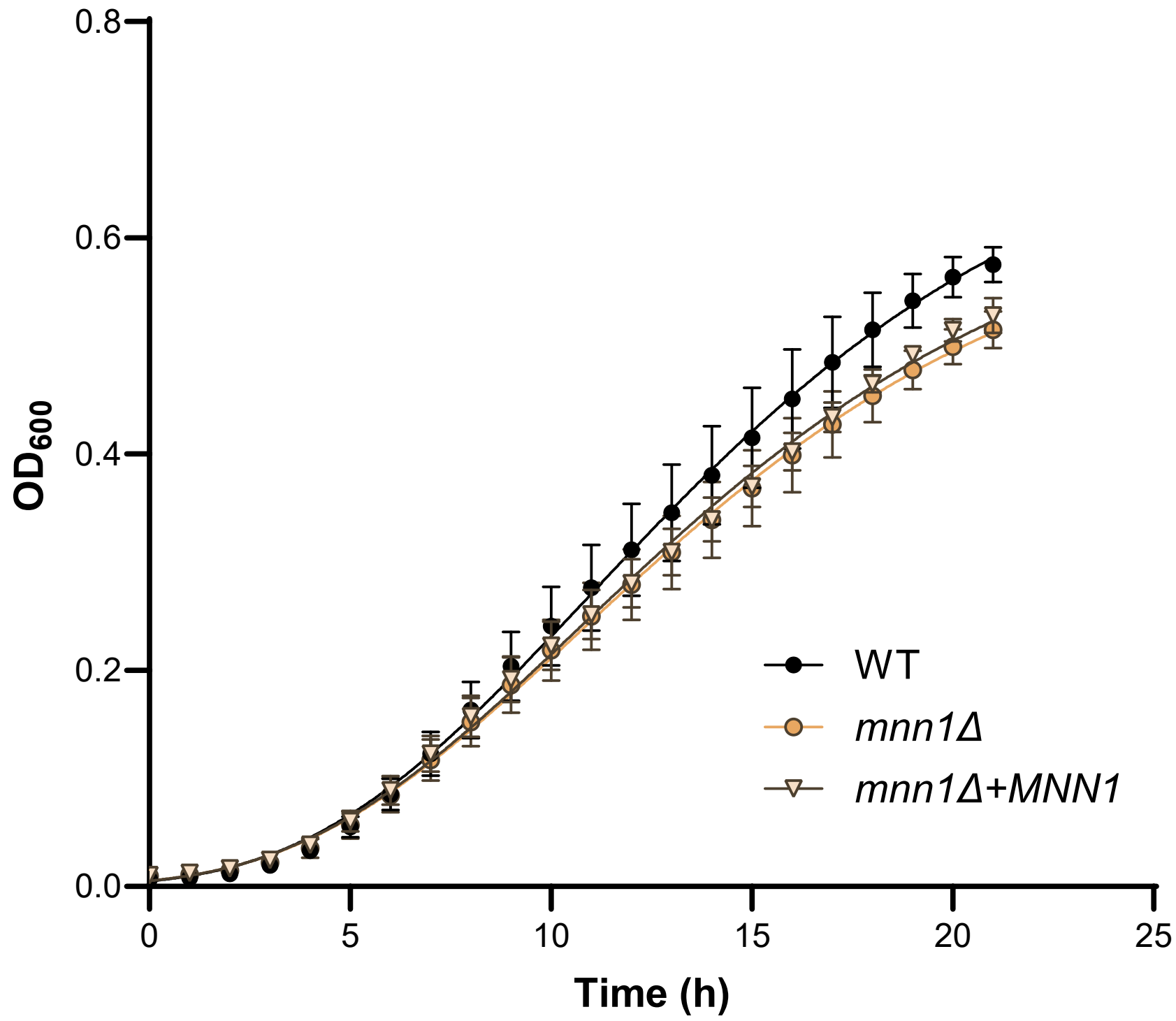

B

Best-fit values	WT	*mnn1Δ*	*mnn1Δ+MNN1*
**YM**	0.7055	0.6374	0.6866
**Y0**	0.003685	0.005161	0.007218
**K**	0.1554	0.1472	0.1359
**1/K**	6.433	6.792	7.359

Figure S2. *C. auris mnn1Δ* strain has similar growth curve compared to *C. auris* WT. (**A**) *C. auris* cells in RPMI supplemented with 2% fetal bovine serum (FBS) were added in triplicate to a clear 96-well microtiter plate ($2x10^5$ cells per well). The plate was incubated for 21 hours at 30°C with absorbance at 600 nm measured every hour. Values adjusted to subtract the absorbance of blank media only controls. Mean with SEM shown for each time point (n=3). Each dataset fit to a nonlinear regression Gompertz growth curve in Graphpad Prism v10.4.2 (calculated regression values shown in (**B**)). No significant difference between k values for each curve.

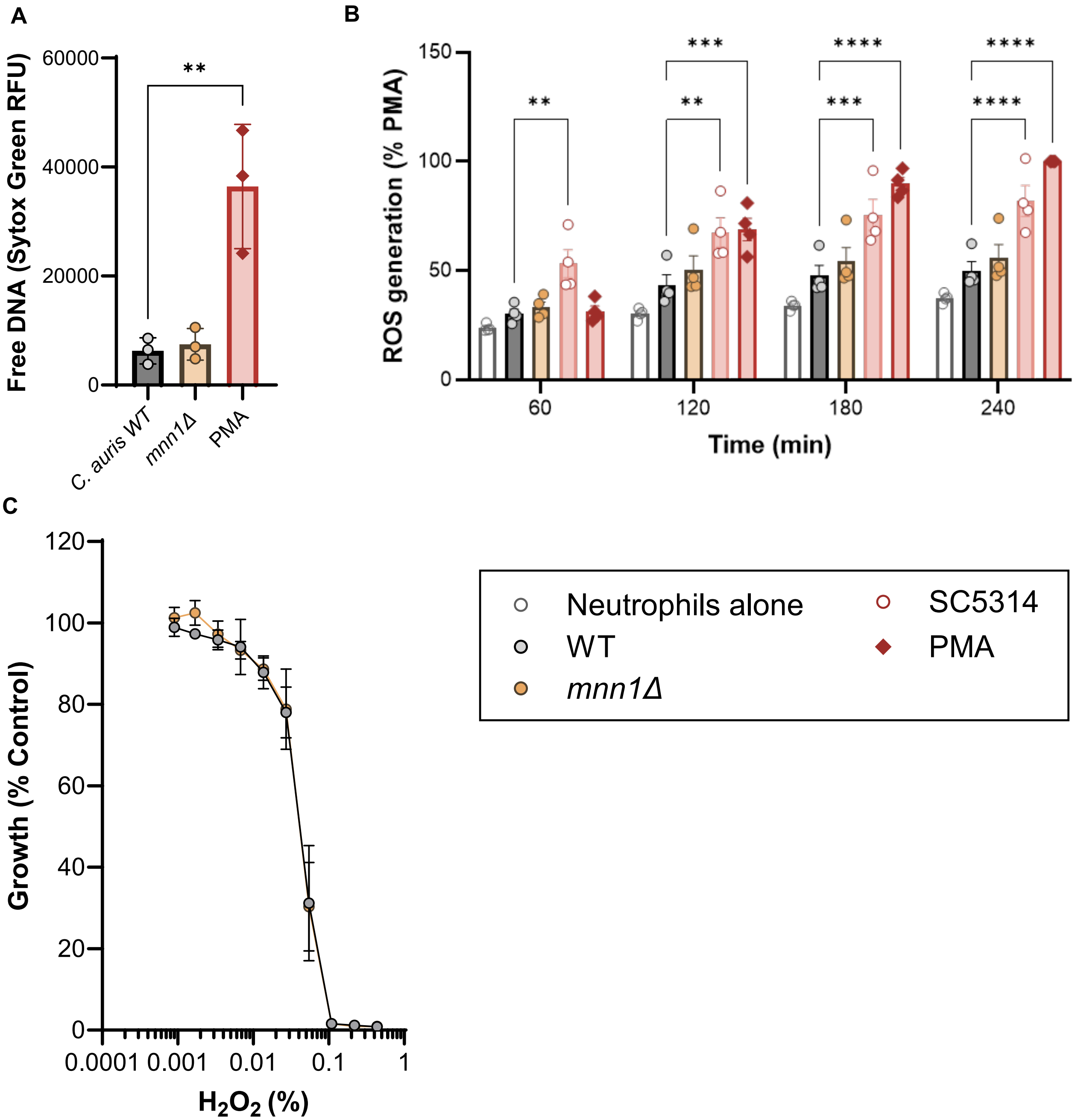

Figure S3. Neutrophils do not produce NETs or ROS in response to *mnn1Δ*. (**A**) Individual *C. auris* strains were incubated for 4 h with human neutrophils before Sytox Green was added to quantify extracellular DNA by fluorescence (500/528nm) in a microplate reader. A subset of wells were given PMA as a positive control for NET formation. (**B**) Human neutrophils were coincubated with *C. auris* mutants for 4 h. CM-H_2DCFDA was included in wells to quantify production of intracellular ROS via fluorescence (495/527 nm) measured by microplate reader. PMA was included in a subset of wells for positive control for ROS generation. (**C**) Sensitivity to hydrogen peroxide was determined by microdilution and incubation for 16h at 30°C. For all experiments n≥3 biological replicates. Mean with SEM shown. *p<0.05, **p<0.01, ***p<0.001, ****p<0.0001 by (A) one-way ANOVA with Holm-Sidak multiple comparisons to *C. auris* WT or (B and C) two-way ANOVA with Holm-Sidak multiple comparisons to *C. auris* WT .

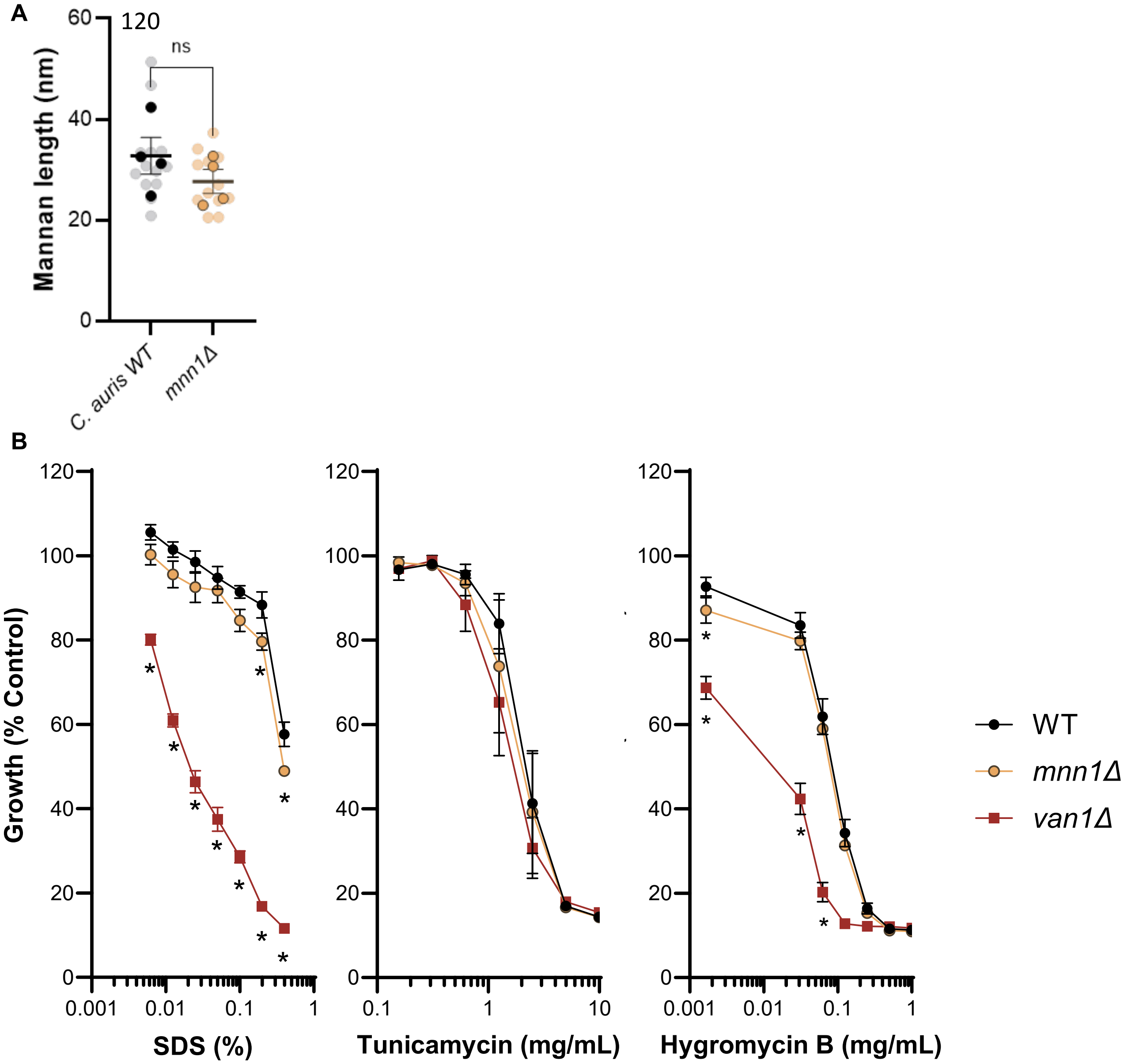

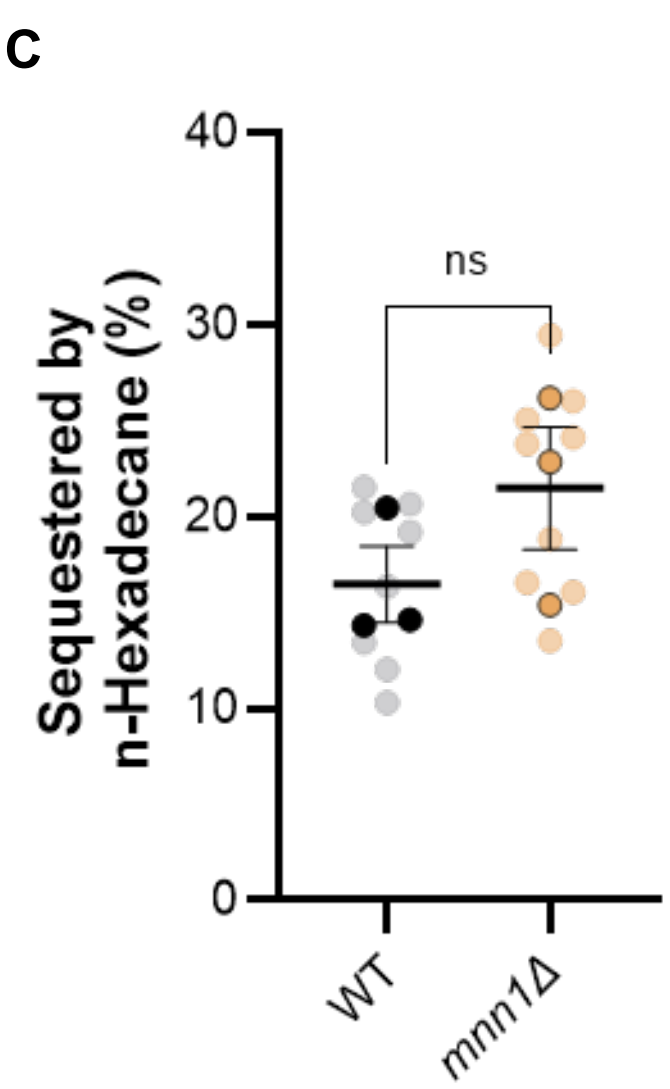

Figure S4. No major differences in mannan length, cell wall stability, or attachment to hydrocarbons between *mnn1Δ* and the parent strain. (**A**) Lengths of cell wall mannan layer from TEM images were manually measured using Fiji. Four different TEM images were utilized for each strain, with three separate length measurements per image. Analyzed by two-tailed unpaired t-test, ns = not significant. (**B**) The sensitivities of the wild type (black circles) and *mnn1Δ* null mutant (yellow circles) to cell wall perturbing agents were determined by absorbance at 600nm after 16h exposed to microdilutions of stressors. The *van1Δ* mutant (red squares) was included as a positive control. (**C**) Proportion of cells sequestered out of the aqueous phase by n-hexadecane during the microbial attachment to hydrocarbons (MATH) assay. n≥3 biological replicates. Mean with SEM shown. *p<0.05 by (A, C) two-tailed unpaired t-test, or (B) two-way ANOVA with Holm-Sidak multiple comparisons to *C. auris* wildtype.

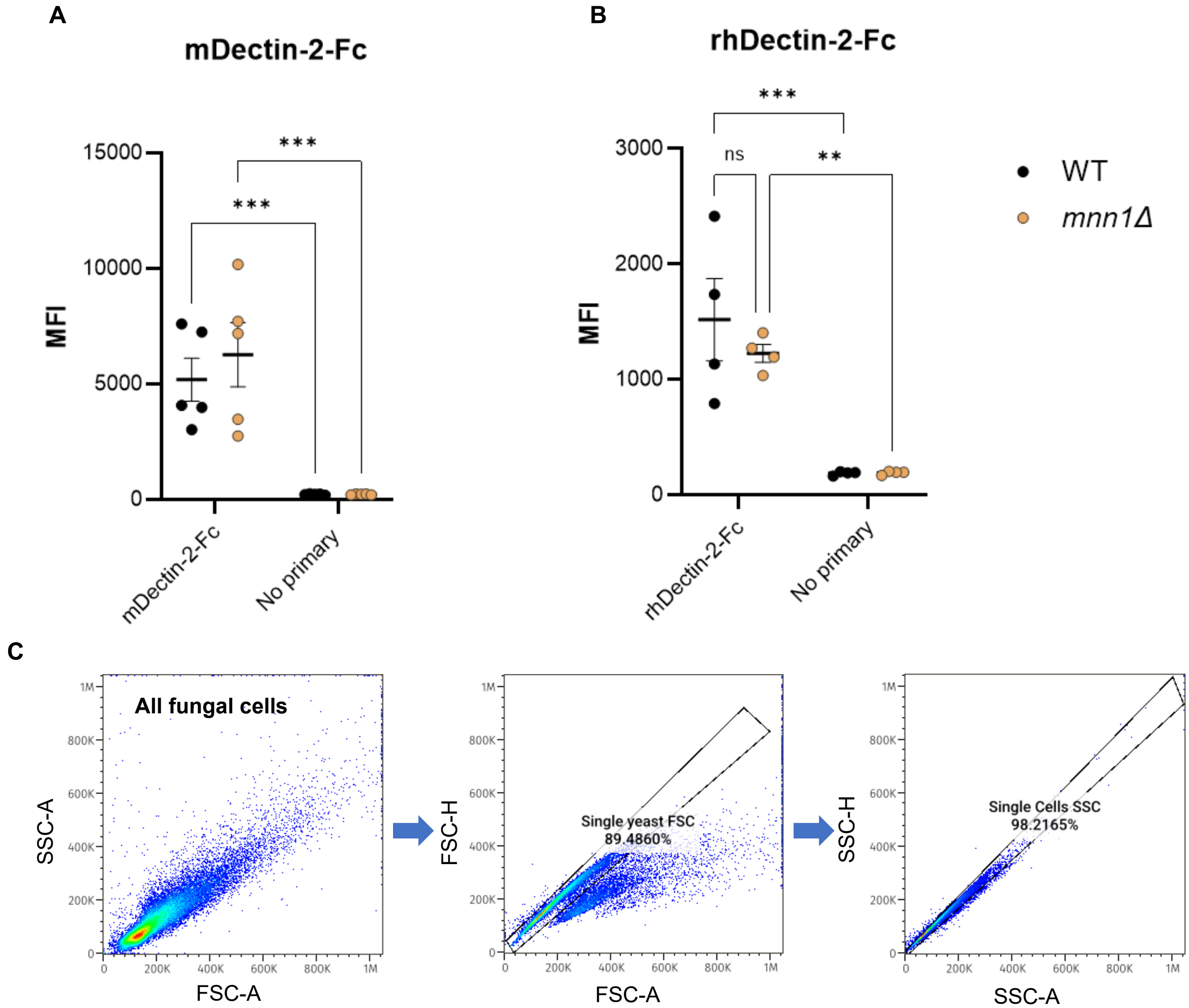

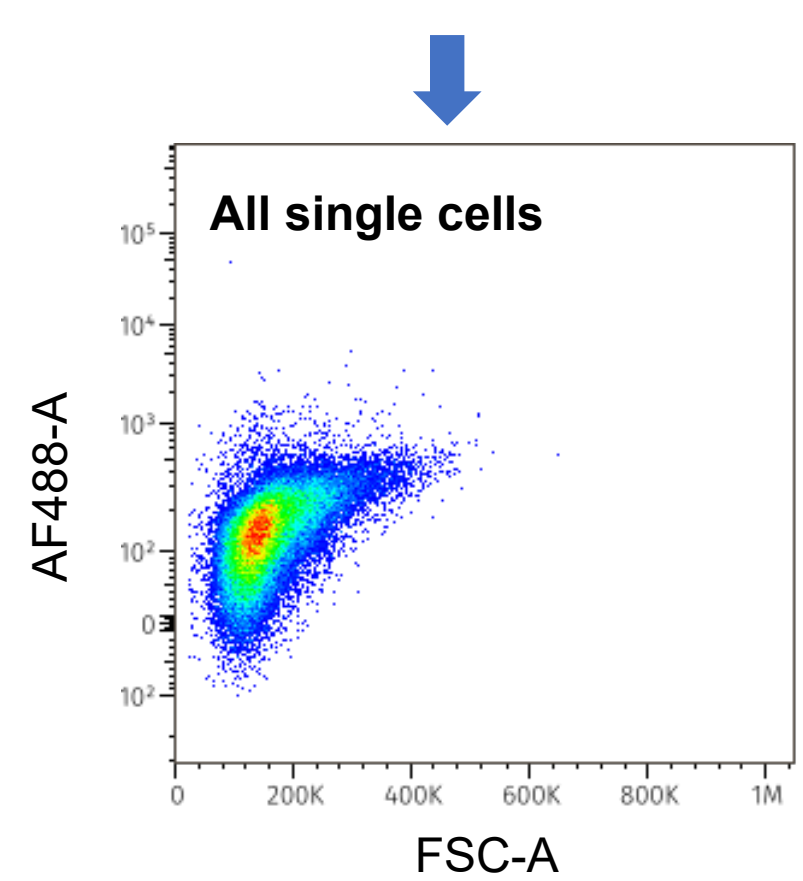

Figure S5. Murine and Human recombinant Dectin-2 binds live *C. auris* WT and *mnn1Δ* fungal cells. Soluble recombinant Dectin-2 (mDectin-2 (**A**) or rhDectin-2 (**B**) each conjugated to rhFc fragment) was incubated with fungal cells for 1 hour on ice, and buffer only treated cells served as no primary controls. All samples were then stained with Alexa Fluor 488-conjugated anti-human IgG Fc antibody and fixed with formaldehyde. Fluorescence was quantified via flow cytometry. Median fluorescence intensity (MFI) of all the single cells was calculated for each experimental day. (**C**) The flow cytometry gating strategy to select for single fungal cells. n≥4. $^{**}p<0.01$, $^{***}p<0.001$ by two-way ANOVA with uncorrected Fisher's LSD. ns=not significant.

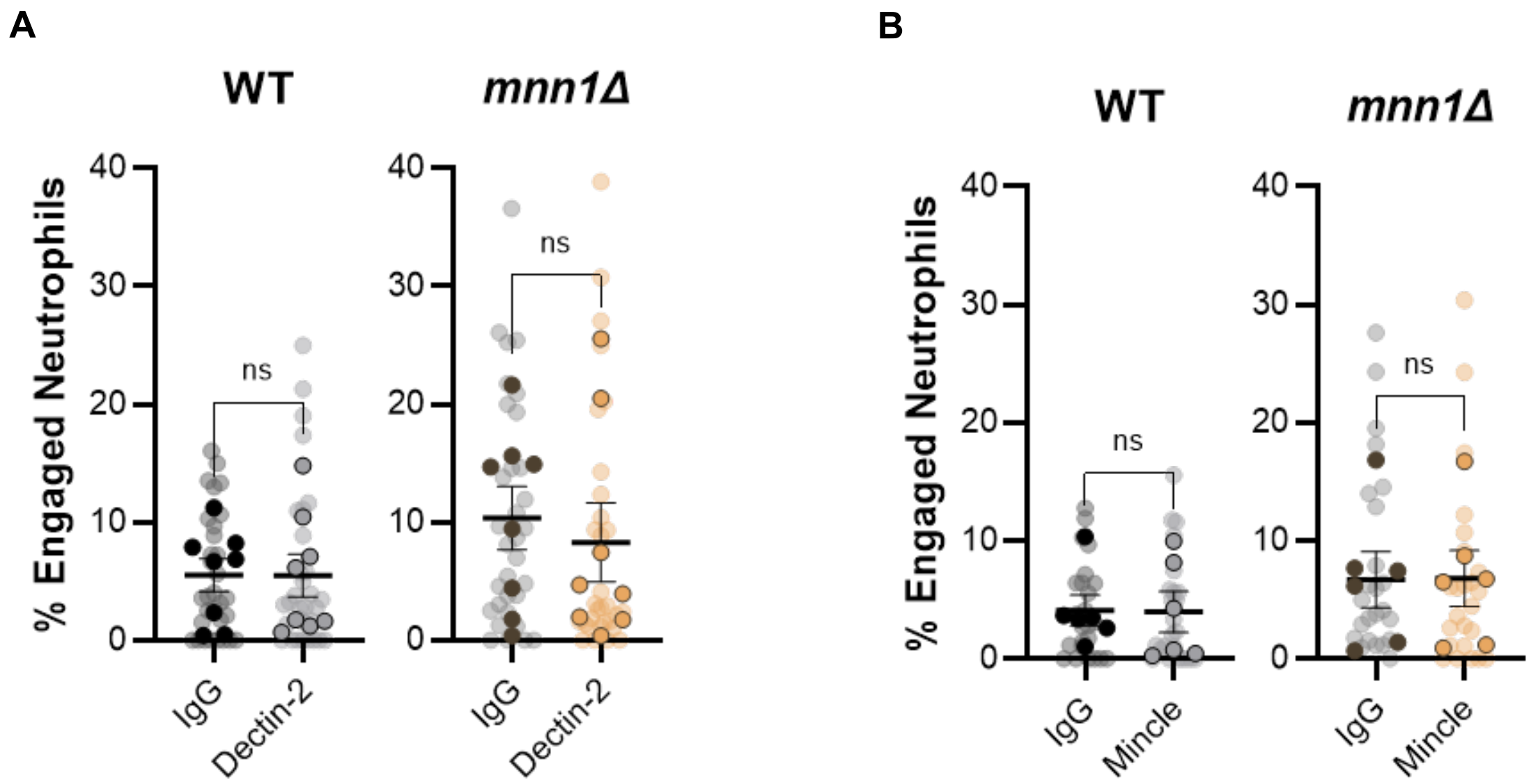

Figure S6. The Fab blocking data before outlier analysis. (**A,B**) Prior to the phagocytosis assay, primary human neutrophils were treated with Fabs generated from (A) mouse IgG2B or anti-human-Dectin-2 antibodies at approximately 5 µg/mL or (B) mouse IgG2B or anti-human-Mincle antibodies at approximately 1 µg/mL for 30 minutes at room temperature. After Fab treatment, neutrophils were co-incubated with calcofluor white-stained fungal cells for 1h. Microscopy images were taken promptly after this incubation. Neutrophil engagement was quantified from microscopy images as described above. (A,B) demonstrates the data without outliers removed, and the data post-outlier analysis is shown in Figure 4B-C. n≥6, ns = not significant by paired t-test to the isotype control.

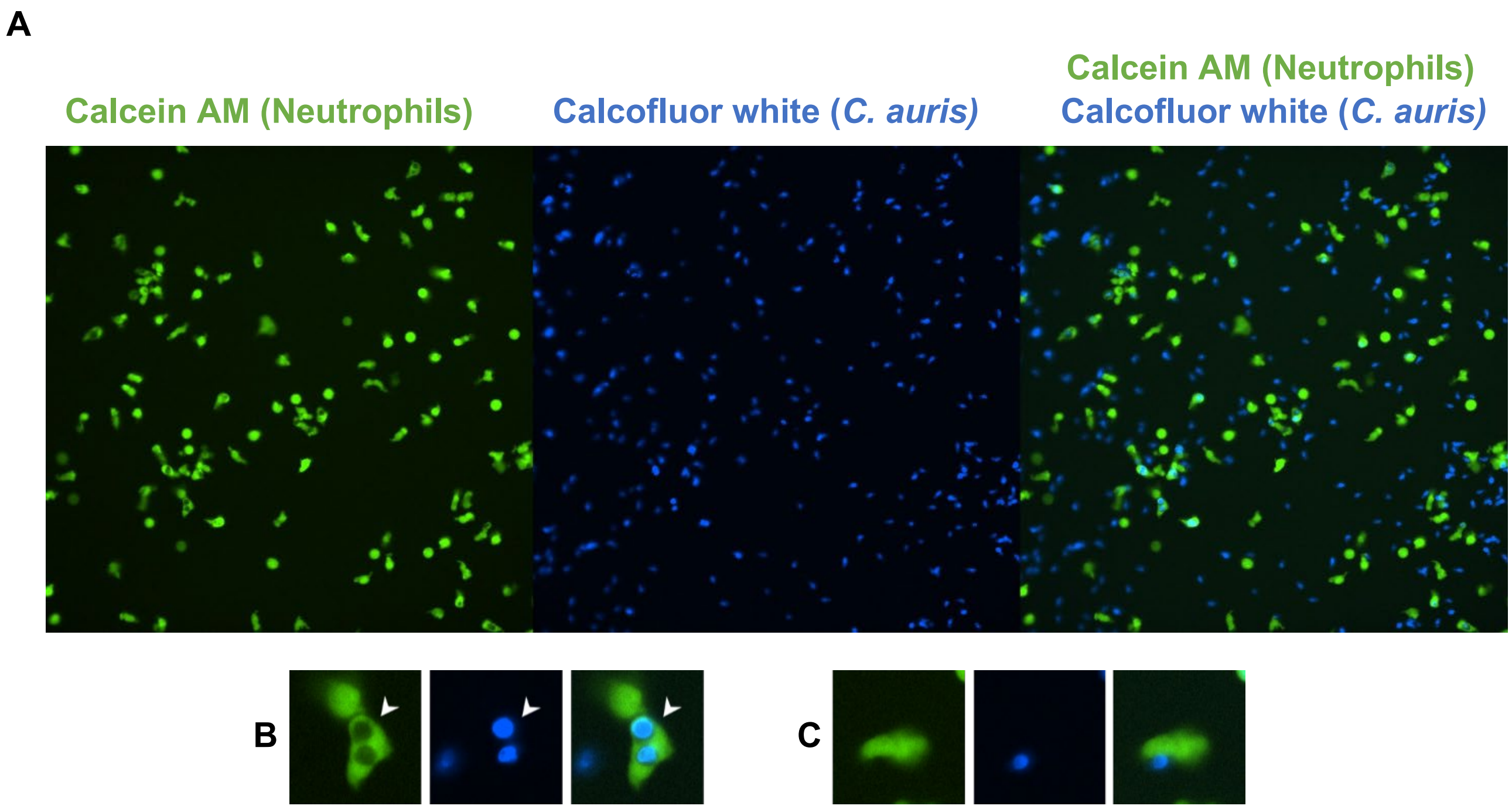

Supplemental S7. Manual image analysis for quantification of neutrophil engagement as a measure of phagocytosis. (**A**) Green and blue channels of an example image. Four to five images were captured per sample on each experimental day. All neutrophils in the field of view were counted with the DIC image and calcein AM staining (green fluorescence). Neutrophils engaging yeast were counted by identifying neutrophils that appeared to be engulfing yeast (calcein AM and calcofluor white stain co-location with neutrophil fluorescence surrounding the fungal cell). (**B**) shows an example of what would be considered a neutrophil engaging yeast. (**C**) demonstrates an example that would not be counted as an engaged neutrophil, simply a yeast near a neutrophil.

References

1. Horton MV, Johnson CJ, Zarnowski R, Andes BD, Schoen TJ, Kernien JF, Lowman D, Kruppa MD, Ma Z, Williams DL, Huttenlocher A, Nett JE. 2021. Candida auris Cell Wall Mannosylation Contributes to Neutrophil Evasion through Pathways Divergent from Candida albicans and Candida glabrata. mSphere 6:e0040621.
2. El Gueddari NE, Rauchhaus U, Moerschbacher BM, Deising HB. 2002. Developmentally regulated conversion of surface-exposed chitin to chitosan in cell walls of plant pathogenic fungi. New Phytologist 156:103–112.
3. Johnson CJ, Davis JM, Huttenlocher A, Kernien JF, Nett JE. 2018. Emerging Fungal Pathogen Candida auris Evades Neutrophil Attack. mBio 9:e01403–18.
4. Santana DJ, Anku JAE, Zhao G, Zarnowski R, Johnson CJ, Hautau H, Visser ND, Ibrahim AS, Andes D, Nett JE, Singh S, O'Meara TR. 2023. A Candida auris-specific adhesin, Scf1, governs surface association, colonization, and virulence. Science 381:1461–1467.
5. Bates S, MacCallum DM, Bertram G, Munro CA, Hughes HB, Buurman ET, Brown AJP, Odds FC, Gow NAR. 2005. Candida albicans Pmr1p, a Secretory Pathway P-type Ca2+/Mn2+-ATPase, Is Required for Glycosylation and Virulence*. Journal of Biological Chemistry 280:23408–23415.
6. Bruno M, Kersten S, Bain JM, Jaeger M, Rosati D, Kruppa MD, Lowman DW, Rice PJ, Graves B, Ma Z, Jiao YN, Chowdhary A, Renieris G, van de Veerdonk FL, Kullberg BJ, Giamarellos-Bourboulis EJ, Hoischen A, Gow NAR, Brown AJP, Meis JF, Williams DL, Netea MG. 2020. Transcriptional and functional insights into the host immune response against the emerging fungal pathogen Candida auris. Nat Microbiol 5:1516–1531.
7. Graus MS, Wester MJ, Lowman DW, Williams DL, Kruppa MD, Martinez CM, Young JM, Pappas HC, Lidke KA, Neumann AK. 2018. Mannan Molecular Substructures Control Nanoscale Glucan Exposure in Candida. Cell Reports 24:2432–2442.e5.
8. Nogueira F, Istel F, Jenull S, Walker L, Gow N, Lion T. 2017. Quantitative Analysis of Candida Cell Wall Components by Flow Cytometry with Triple-Fluorescence Staining. Journal of Microbiology and Modern Techniques 2.
9. Lockhart SR, Etienne KA, Vallabhaneni S, Farooqi J, Chowdhary A, Govender NP, Colombo AL, Calvo B, Cuomo CA, Desjardins CA, Berkow EL, Castanheira M, Magobo RE, Jabeen K, Asghar RJ, Meis JF, Jackson B, Chiller T, Litvintseva AP. 2017. Simultaneous Emergence of Multidrug-Resistant Candida auris on 3 Continents Confirmed by Whole-Genome Sequencing and Epidemiological Analyses. Clin Infect Dis 64:134–140.
